# Supplementary material for: An Innovative Curriculum to Empower Trainees and Faculty to Address Patient-Initiated Identity-Based Misconduct in the Clinical Learning Environment
Source: MedEdPORTAL. 2026 Apr 9;22:11591. doi: 10.15766/mep_2374-8265.11591 (PMC13061878; doi:10.15766/mep_2374-8265.11591)
Supplement: Supplementary file 1 — I-RESPOND Toolkit.docxFacilitator Guide.docxEvaluations.docxPresentation.pptxScenario Scripts.docx [file mep_2374-8265.11591-s001.zip › B. Facilitator Guide.docx]

This guide is intended for faculty facilitators preparing to deliver the I-RESPOND workshop. It provides background context, step-by-step instructions, and supplemental resources to ensure consistent delivery of the curriculum across settings. The first section outlines essential materials and core content that must be reviewed before leading a session. Additional sections include expanded background literature, suggested discussion prompts, and optional practice scenarios for facilitators who wish to adapt or deepen the training. Facilitators should review the “Core Content” before leading their first workshop and may use the supplemental sections as reference materials. This guide was designed to support both initial facilitator training (e.g., in a 4-hour train-the-trainer session) and as an on-hand resource during workshop delivery.

**Train the Trainer session for the workshop: *Responding to Patient-Initiated Identity-Based Harassment: An Innovative Curriculum to Empower Residents and Faculty to Address Discrimination and Harassment in the Clinical Training***

Table of Contents

[**Facilitator Instructional Guide Overview i**v](#_bookmark0)

**Slide 1– Introduction/Title** 1

**Slide 2: Learning Objectives** 2

**Slide 3: Discussion Guidelines** 3

**Slide 4: Overview** 4

**Slide 5: Discrimination & Harassment of Medical Trainees** 5

**Slide 6: Introduction – Identity Groups** 6

**Slide 7: Introduction – Discrimination and Harassment** 7

**Slide 8: Patient and Visitor Comments** 9

**Slide 9: Audience Poll #1** 10

**Slide 10: Harassment & Discrimination in Healthcare** 12

**Slide 11: Article entitled “Discrimination, Abuse, Harassment, and Burnout in Surgical Residency Training**

**.** 13

**Slide 12: Harassment of the medical trainee** 14

**Slide 13: Impact of Harassment** 15

**Slide 14: What are Barriers to Responding to patient harassment?** 17

**Slide 15: Tools for Responding to Patient‐Initiated Harassment** 19

**Slide 16: Audience Poll #2** 20

**Slide 17: Current harassment training is insufficient** 22

**Slide 18: General Considerations** 23

**Slide 19: Response Toolkit** 25

**Slide 20: Use “I” Statements** 26

**Slide 21: Repeat and Clarify Statement** 27

**Slide 22: Emphasize Shared Goals** 28

**Slide 23: Set Boundaries** 29

**Slide 24: Patient Actions rather than Person** 30

**Slide 25: Offer and Alternative** 31

**Slide 26: Separate Intent from Impact** 32

**Slide 27: Don’t Use Humor** 33

**Slide 28: Practice Skills** 34

**Slide 29: Scenario 1 (Video 1)** 35

**Slide 30: How would you respond? – Scenario 1** 36

**Slide 31: Scenario 1 (Video 2) Putting it all together** 38

**Slide 32: What if you witness harassment?** 39

**Slide 33: Bystander** 40

**Slide 34: If you Observe Harassment of Trainee or Colleague (slide 1)** 41

[**Slide 35: If you observe harassment of trainee or colleague (slide 2)** 42](#_TOC_250003)

**Slide 36: Scenario 2** 43

[**Slide 37: How would you respond? – Scenario 2** 44](#_TOC_250002)

**Slide 38: Wrap‐Up** 45

**Slide 39: Graduate Medical Education Guidelines** 46

**Slide 40: Key Points** 48

**Slide 41: What is one skill from this workshop you want to remember?** 49

**Slide 42: We Stand Together** 50

**Slide 43: Questions** 51

[**Slide 44: Thank you** 52](#_TOC_250001)

[**Slide 45: Additional Content** 53](#_TOC_250000)

**Slide 46: Discriminatory Patient Preference Requests** 54

##### Responding to Patient‐Initiated Identity‐Based Harassment: An Innovative Curriculum to Empower Residents and Faculty to Address Discrimination and Harassment in Clinical Training

Facilitator Instructional Guide Overview

##### Overview:

Harassment based on identity (race, ethnicity, gender, etc.) is highly prevalent during medical training, and patients are a common source. Forms of harassment from patients can range from rude and disruptive behavior with inappropriate comments, or innuendos to more egregious verbal and physical abuse. As frontline workers, medical learners are in uniquely vulnerable positions and more likely to be targets of patient harassment. Harassment negatively affects learners’ mental health, work performance, and ability to provide optimal patient care. Training on communication skills and strategies for addressing harassment from patients is lacking. A proactive and intentional approach is needed to ensure that medical learners and supervisors are adequately prepared to respond to harassment.

This workshop is intended to be given to individuals who work within a healthcare setting. Recommend two facilitators to implement the workshop. However, the workshop can be done with just 1 facilitator. When facilitating with two individuals, would recommend alternating the slides. Also, co-lead large group discussions.

##### Workshop Objectives:

1. Define and recognize the various forms of identity-based patient misconduct.
2. Discuss the prevalence and implications of identity-based patient misconduct in the clinical learning environment.
3. Identify barriers to responding to incidents of misconduct.
4. Learn and implement at least 3 communication strategies for responding to patient misconduct.
5. Outline the role of bystanders in observing and addressing misconduct by patients.

##### Audience:

Medical students, House staff (residents, fellows), and faculty

##### Workshop Handouts and Materials

- 1. PowerPoint presentation
  2. I-RESPOND Toolkit for Addressing Patient-Initiated Identity-Based Harassment - (double sided)
     1. For virtual presentations:
        1. Handout 1 (front) & Handout 2 (back)
  3. Practice Skills Small Group Handout
  4. Evaluations (to be sent electronically after the workshop)

##### Slide Instructions

On the slides, italicized font denotes facilitator notes NOT meant to be read out loud. Non-italicized font denotes suggested narratives meant to be read out aloud.

*Prior to the workshop:*

- For a live presentation, it is recommended that workshop handouts (I-Respond and Practice skills) are printed and given to participants during the presentation. Recommend printing the handouts double-sided. Pens should be available for participants to fill out the evaluation forms if not sent electronically. Consider having tables and chairs arranged in order to facilitate small group discussion for 2-3 participants.
- For virtual presentations, recommend sending the I-Respond and Practice skills handout files in the chat during the workshop.

***For live presentations:***

- - Arrange for polls to be set up ahead of time.
    - **Audience Poll #1 (slide 9):** *Poll Question and Answer options:*

1. *What is your personal experience with identity-based harassment? I have experienced it myself.*

*I have observed it*

*I have both experienced it myself and observed it None of these apply to me.*

1. *Who was the source of the harassment behavior? Check all that apply. Supervisors*

*Fellow colleagues Support staff Administration*

*Patient or patient’s family/visitors*

- - **Audience Poll #2 (slide 16)**: *Poll Question and Answer options:*

1. *Have you previously received training on techniques for responding to patient harassment?*

*Yes No*

1. *If you experience harassment or observe harassment of a colleague. How prepared do you feel to respond to the harassment?*

*Very prepared Slightly prepared Neutral*

*Slightly unprepared Very unprepared*

***Suggested Instructions for virtual presentations on Zoom:***

***Prior to Zoom Session:***

- Arrange for polls to be set up ahead of time.
  - **Audience Poll #1 (slide 9):** *Poll Question and Answer options:*

1. *What is your personal experience with identity-based harassment? I have experienced it myself.*

*I have observed it*

*I have both experienced it myself and observed it None of these apply to me.*

1. *Who was the source of the harassment behavior? Check all that apply. Supervisors*

*Fellow colleagues Support staff Administration*

*Patient or patient’s family/visitors*

- - **Audience Poll #2 (slide 16)**: *Poll Question and Answer options:*

1. *Have you previously received training on techniques for responding to patient harassment?*

*Yes No*

1. *If you experience harassment or observe harassment of a colleague. How prepared do you feel to respond to the harassment?*

*Very prepared Slightly prepared*

*Neutral*

*Slightly unprepared Very unprepared*

- Arrange for two break out groups of 2-3 people for 4 minutes to be set up ahead of time
- Ensure that someone is available to help manage the chat if you are presenting alone
- Ensure that someone is available to help drop handout files into the chat
  - Make “File transfer” is enabled under “Settings” in zoom

##### During Zoom Session:

- When sharing your screen be sure to select the share sound check box
- Follow zoom actions as detailed below

##### Suggested Timeline:

This workshop should be approximately 60 minutes as summarized below. Work shop can be adapted to 90 minutes.

For live presentation:

| **Slide numbers** | **Time** | **Content discussed** | **Actions** |
| --- | --- | --- | --- |
| Slides 1-4 | 4 min  (0-04) | Introductions, objectives, discussion guidelines, and workshop overview |  |
| Slides 5-7 | 3 min  (04-07) | Define identity groups and define Key Concepts |  |
| Slides 8- 9 | 4 min  (07-11) | Example quotes and audience reflections in chat, and audience Poll #1 | #9: Audience Poll  Have participants open survey link by scanning the QR code with the Camera App on their phone.  Once everyone has completed the survey, the presenter/facilitator can click on the “Results” link on the slide to view the results. Then return to the PowerPoint |
| Slide 10-14 | 8 min  (11-19) | Review prevalence and impact, discuss barriers to  responding to harassment and request answers in the chat |  |

|  |  |  |  |
| --- | --- | --- | --- |
| Slide 15-18 | 3 min  (19-22) | Assess previous communication training on responding to harassment (poll #2) and comfort level and discuss lack of formal training. | #16: Audience Poll  Have participants open survey link by scanning the QR code with the Camera App on their phone.  Once everyone has completed the survey, the presenter/facilitator can click on the “Results” link on the slide to view the results. Then return to the  PowerPoint |
| Slide 19- 27 | 8 min  (22-30) | Discuss approaches to responding and I-RESPOND toolkit | #20: Pass out the I-RESPOND handout |
| Slide 28 -30 | 10 min  (30-40) | Watch video #1 and practice Skills in small groups and discuss with the large group | #28 Pass out the “Practice Skills Small Group Handout” and encourage participants to refer to it during their small group discussion.  4-minute break out groups of 2-3 people. |
| Slide 31- 35 | 6 min  (40-46) | Review role of bystanders in responding |  |
| Slides 36- 37 | 10 min  (46-56) | Watch clinical scenario video #2, small groups and large group discussion, watch another version of scenario #2 and large group discussion | #37: Prior to breakout groups: Have participants refer to the “Practice Skills Small Group Handout” for questions to answer during this small group discussion  4-minute break out groups of 2-3 people. |
| Slide 38 - 42 | 4 min  (56-60) | Wrap up |  |
| Slide 43 - 44 |  | Questions |  |

For virtual (zoom) presentation:

| **Slide numbers** | **Time** | **Content discussed** | **Zoom Actions** |
| --- | --- | --- | --- |
| Slides 1-4 | 4 min  (0-04) | Introductions, objectives, discussion guidelines, and workshop overview | Share Presentation Check share sound box |
| Slides 5-7 | 3 min  (04-07) | Define identity groups and define Key Concepts |  |
| Slides 8- 9 | 4 min  (07-11) | Example quotes and audience reflections in chat, and audience Poll #1 | #8: Ask audience members to post reactions in the chat (2 words). Read out loud.  #9: Audience Poll  Start Zoom Poll # 1. Share  results. Stop share results. Close poll. |
| Slide 10-14 | 8 min  (11-19) | Review prevalence and impact, discuss barriers to responding to harassment and request answers in the chat | #14: Ask participants to add their response to the chat. Read comments out loud. |
| Slide 15-18 | 3 min  (19-22) | Assess previous communication training on responding to harassment (poll #2) and comfort level, and discuss lack of formal training. | #16: Audience Poll  Start Zoom Poll # 2. Share results. Stop share results. Close poll. |
| Slide 19- 27 | 8 min  (22-30) | Discuss approaches to responding and I-RESPOND toolkit | #20: Drop I-RESPOND handout in zoom chat |
| Slide 28 -30 | 10 min  (30-40) | Watch video #1 and practice Skills in small groups and discuss with the large group | #28: Prior to break out groups: Add the “Practice Skills Small Group Handout” to the chat or post the following below in |

|  |  |  | chat so groups can reference during breakouts.  **Patient comment**: *“Oh sweetheart, how about if you just climb into this bed with me.”*  **Question**: **How would you respond?**  ***Practice how you would respond if you were the resident/student. It is important that each of you rehearse how you would respond to the inappropriate patient comment in your own words. Refer to the “I- RESPOND Toolkit. Also, consider discussing which responses may be effective or ineffective in this scenario.  4-minute break out groups of 2-3 people. |
| --- | --- | --- | --- |
| Slide 31- 35 | 6 min  (40-46) | Review role of bystanders in responding |  |
| Slides 36- 37 | 10 min  (46-56) | Watch clinical scenario video #2, small groups and large group discussion, watch another version of scenario #2 and large group discussion | #37: Prior to breakout groups: If not done previously on slide 28, add the “Practice Skills Small Group Handout” file to the chat, or you can type the following questions to discuss into chat so groups can reference during breakouts.   1. **What went wrong in this scenario?** 2. **How would you respond differently to the patient?** 3. **How would you support the learner?** |

|  |  |  | 4-minute break out groups of 2-3 people. |
| --- | --- | --- | --- |
| Slide 38 - 42 | 4 min  (56-60) | Wrap up  Questions | #41: Share one skill from this workshop you want to remember? Ask audience to type in chat. Read aloud. |
| Slide 43 -44 |  |  |  |


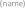

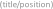


“What to Say”: Responding to Patient-Initiated Misconduct

- Addressing Identity-Based Misconduct in the Clinical Learning Environment

(name) (title/position)

(date)

Slide 1 – Introduction/Title

*Add names, background information of facilitators, and date to slide.*

*During the presentation, when you share your screen, make sure to select****“share sound”***

*Introduction: The facilitators should read the title of the workshop and then introduce themselves to the audience and discuss their roles in their*

*respective institutions. Facilitators may choose to mention they are following the cont ent and guidelines of this peer-reviewed module.*

Hello. Today we will be discussing “What to say”: Responding to Patient- Initiated Harassment –

Addressing Identity-Based Harassment in the Clinical Learning Environment. [Insert facilitators name and title]. This workshop today is a part of a peer-reviewed module.

*Facilitators should give a “trigger waning” to participants so that they are aware of the content that will be discussed in this workshop.*

This talk will include detailed examples and audience discussion of patient-initiated harassment that is focused on health care providers’ identity, such as one’s sex, gender, physical appearance, race, sexual orientation, or age.

*For virtual presentations where handouts were sent electronically, make sure that participants have access to all the handouts. Consider including the handouts in the chat to ensure that participants have the them.*

I would like to make sure that everyone has access to the materials. Therefore, we will attach the handouts to the chat.

Learning objectives

- Define and recognize the various forms of identity-based patient misconduct
- Discuss the prevalence and implications of identity-based patient misconduct in the clinical learning environment
- Identify barriers to responding to incidents of misconduct
- Learn and implement at least 3 communication strategies for responding to patient misconduct
- Outline the role of bystanders in observing and addressing misconduct by patients

Slide 2: Learning Objectives

*Review the learning objectives:*

****By the end of this workshop, you should be able to define and recognize the various forms of identity-based patient misconduct, discuss the prevalence and impact of patient harassment on medical trainees, identify barriers to responding to incidents of harassment, learn and apply at least 3 communication strategies for responding to harassment by patients, and describe the role of bystanders in monitoring and responding to harassment by patients.

### Discussion Guidelines


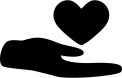

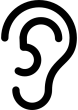

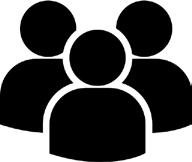


#### Respect Confidentiality

Stories Stay and Lessons Leave

#### Active Listening

Pay attention, Withhold judgment, Reflect, Clarify, Correct gently but do correct, Share

#### Shared Goal

To learn and build on communication skills that will help us provide the best care to our patients

Slide 3: Discussion Guidelines

*This slide is intended to highlight guidelines for Respectful Discussion.*

We want this to be a safe space for candid discussion about an issue that affects our learners and ask that we respect each other’s confidentiality. “Stories Stay and Lessons Leave” In terms of stories some of you have direct experiences, observed scenarios or heard second hand and want to help.

We encourage active listening and ask that when others are sharing, we pay attention, listen with an open mind and hold judgement, reflect on the discussion, ask for clarification when needed, correct gently, but do correct and share experiences and perspectives.

Finally, the goal is to learn and build on communication skills that will help us provide the best care to our patients and an inclusive environment for our team

.

*Reference: 6 key active listening skills (Center for Creative Leadership) https://*[*www.ccl.org/articles/leading-*](http://www.ccl.org/articles/leading-effectively-articles/coaching-others-use-active-listening-) [*effectively-articles/coaching-others-use-active-listening-*](http://www.ccl.org/articles/leading-effectively-articles/coaching-others-use-active-listening-) *skills/*

Active listening icon: Image made by Roundicons from www.flaticon.com, retrieved from https://[www.flaticon.com/free-](http://www.flaticon.com/free-) icon/ear_467717?term=ear&page=1&position=2&origin=search&related_id=467717 on March 2, 2023. Image is in public domain and free for personal and commercial purpose with attribution.

Respect Confidentiality Icon: Image made by Freepik

from www.flaticon.com, retrieved https://[www.flaticon.com/free-icon/hand-with-](http://www.flaticon.com/free-icon/hand-with-) heart_67468?term=hand++with+heart&page=1&position=1&origin=search&related_i d=67468 on March 15, 2023. Image is in public domain and free for personal and commercial purpose with attribution.

Shared Goal Icon: Image made

by Freepik from www.flaticon.com, retrieved https://[www.flaticon.com/free-](http://www.flaticon.com/free-) icon/multiple-users- silhouette_33308?term=people&page=1&position=2&origin=search&related_id=333 08 on March 15, 2023. Image is in public domain and free for personal and commercial purpose with attribution.

Overview

1. Harassment in the clinical learning environment
   1. Effects on medical trainees
   2. Barriers to responding
2. Response toolkit & skills practice
   1. Direct response
   2. Bystander role
3. Wrap-up

Slide 4: Overview

*Provide an Overview of the workshop:*

During our time together we will review harassment in the clinical learning environment. This will include the effects on medical trainees and barriers to responding.

Next, we will be using a RESPONSE toolkit as a guide for different ways to respond to patient-initiated harassment. Then we will have an opportunity to engage in practicing our responses in small groups. This will include direct responses. We will also discuss a bystander’s role in patient-initiated harassment scenarios.

Finally, we will end with a wrap up.

Discrimination & Harassment of Medical Trainees

Slide 5: Discrimination & Harassment of Medical Trainees

*Give an overview of this next part of this training:*

Now we will discuss the various forms of identity-based patient harassment and the prevalence and impact of patient harassment on medical trainees

## Introduction

***“Defined by physical, social, and mental characteristics of individuals”***

Identity Groups

- Race
- Ethnicity
- Gender
- Religion
- Sexual orientation
- Gender identity
- Age
- Disability
- *S*ocioeconomic status
- Geographic location
- Language
- Citizenship status
- Mental health
- Physical appearance

Slide 6: Introduction – Identity Groups

*Provide a definition of Identity Groups:*

Social identity groups are usually defined by some physical, social, and mental characteristics of individuals. Examples of social identities are race, ethnicity, gender, religion, sexual orientation, gender identity, age, socioeconomic status, geographic location, language, citizenship status, mental health, physical appearance. An individual’s social identity may designate who they are based on the groups to which they belong.

Reference:

https://[www.northwestern.edu/searle/initiatives/diversity-equity-inclusion/social-](http://www.northwestern.edu/searle/initiatives/diversity-equity-inclusion/social-) identities.html

Identity Groups Icon: Image made by Freepik from www.flaticon.com, retrieved https://[www.flaticon.com/free-](http://www.flaticon.com/free-) icon/toilet_66886?term=men+and+women&page=1&position=7&origin=search&rela ted_id=66886 on March 15, 2023. Image is in public domain and free for personal and commercial purpose with attribution.


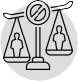


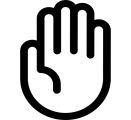

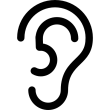


 
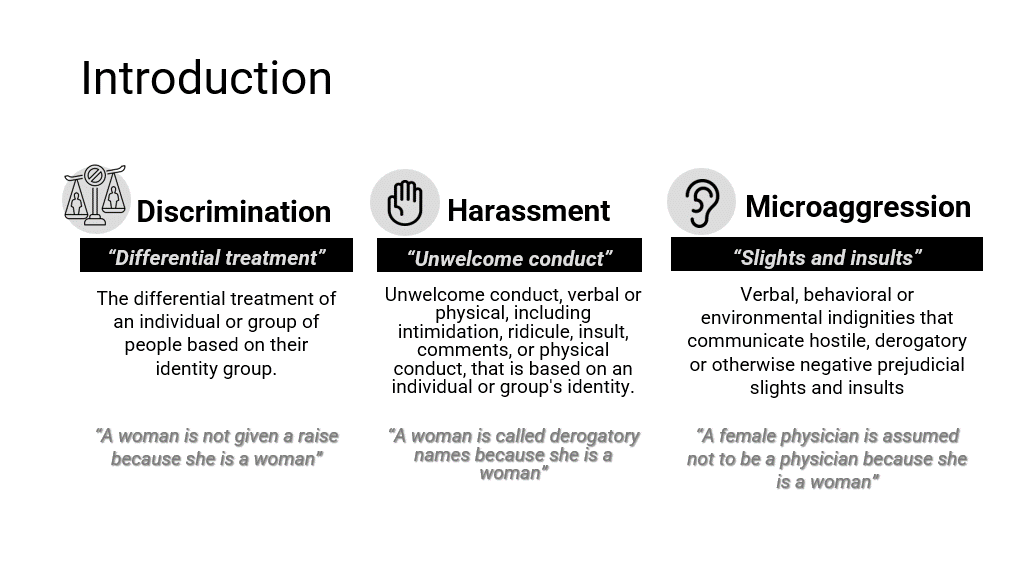


Slide 7: Introduction – Discrimination and Harassment


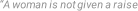

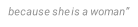

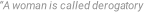

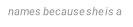

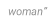

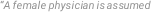

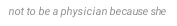

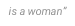


*Give definitions of discrimination and harassment:*

The US Equal Employment Opportunity Commission has provided definitions for Discrimination and Harassment. Discrimination is the differential treatment of an individual or group of people based on their identity group. Discrimination is **commonly viewed through the lens of race and ethnicity but can occur across a broad range of dimensions or identity groups as we saw in the previous slide which includes** characteristics historically linked to discrimination or exclusion. An example of discrimination includes a woman not given a raise because she is a woman.

Harassment involves conduct, verbal or physical, including intimidation, ridicule, insult, that is based on an individual’s identity, when the behavior can reasonably be considered to adversely affect the work environment. An example includes a woman is called derogatory names because she is a woman.

Harassing conduct can range from actions that may not directly impact a provider’s ability to practice (e.g. comments about appearance, attractiveness, inappropriate jokes, etc) all the way to severely disruptive conduct (e.g. threats of physical assault or violence) that makes it impossible for a provider to continue to be the best physician they can be.

*Highlight how other forms of unwelcome conduct, like microaggressions, which can come from patients and visitors and have a significant impact.*

Soon we will be discussing the impact of patient initiated harassment. However, it is important to note

that other forms of unwelcome conduct such as microaggressions, can have a significant impact. Microaggressions include verbal, behavioral, or environmental indignities, whether intentional or unintentional, that communicate hostile, derogatory or otherwise negative prejudicial slights and insults toward any individual or group, particularly culturally marginalized individuals and groups.

Reference: https://[www.hopkinsmedicine.org/news/articles/bullying-](http://www.hopkinsmedicine.org/news/articles/bullying-) microaggression-and-other-terms

Discrimination Icon: Image made by GOWI from www.flaticon.com, retrieved https://[www.flaticon.com/free-](http://www.flaticon.com/free-) icon/bias_7194938?term=discrimination&page=1&position=5&origin=search&relate d_id=7194938 on June 6, 2023. Image is in public domain and free for personal and commercial purpose with attribution.

Harassment Icon: Image made by Freepik from www.flaticon.com, retrieved https://[www.flaticon.com/free-](http://www.flaticon.com/free-) icon/hand_3898664?term=stop+hand&page=1&position=4&origin=search&related_i d=3898664 on March 15, 2023. Image is in public domain and free for personal and commercial purpose with attribution.

Microaggression icon: Image made by Roundicons from www.flaticon.com, retrieved from https://[www.flaticon.com/free-](http://www.flaticon.com/free-) icon/ear_467717?term=ear&page=1&position=2&origin=search&related_id=467717 on March 2, 2023. Image is in public domain and free for personal and commercial purpose with attribution


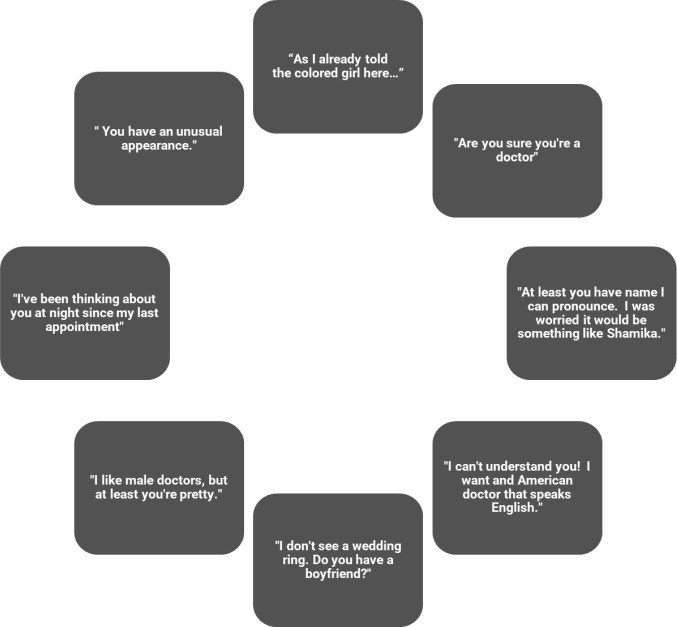

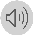

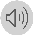

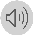

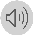

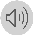

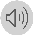

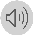

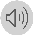


**Design created by Aisha Jamison**

Slide 8: Patient and Visitor Comments

*This slide provides examples of several patient and visitor comments made to medical trainees at the University of Iowa Hospitals and Clinics:*

In the clinical learning environment, trainees experience unwelcome and harassing comments from

patients. Here we highlight several patient and visitor comments that medical trainees have experienced at the University of Iowa Hospitals and Clinics.

*Play the audio recording (press forward once and comments/audio will appear automatically) or read the comments on the slides one by one. [Make sure to check share sound when sharing your screen on zoom].*

*Reactions:*

What are some initial reactions to these patient comments?

*For virtual presentation:* In the chat post 2 words or less and give your reflections.

*Read the commentsout loud from the chat. Feel free to share your own personal or observed experience of harassment.*

# Audience Poll

**Add QR Code Here**

1. Open your Camera App.
2. Aim your camera at the QR code and make sure it is in focus.
3. The QR code should automatically be scanned.
4. Tap on the pop-up text that appears.
5. You will be brought to the QR code link/information.

Slide 9: Audience Poll #1

*In order to assess experiences with harassment, the following poll questions below should be answered by the participants. You can create a poll through Microsoft forms, google forms, etcs.*

*Would recommend adding a QR code to the slide so that participants are able to quickly access the survey.*

*Poll Question and Answer options.*

1. *What is your personal experience with identity-based harassment?*
   - *I have experienced it myself.*
   - *I have observed it*
   - *I have both experienced it myself and observed it*
   - *None of these apply to me.*
2. *Who was the source of the harassment behavior? Check all that apply.*
   - *Supervisors*
   - *Fellow colleagues*
   - *Support staff*
   - *Administration*
   - *Patient or patient’s family/visitors*

*Read the following to the participants:*

We have a couple poll questions we would like you to answer. What is your personal experience with identity-based harassment and who was the source of the harassment behavior? Please open the camera app on your phone.

Aim the camera at the QR code and make sure it is in focus. The QR code should be automatically scanned. Tap on the pop up text that appears on your screen. You will then be brought to the survey link. Please fill out this survey.

*You can create a Results link on your slide that can take participant to a website with the poll answers. Review the poll results with the participants.*


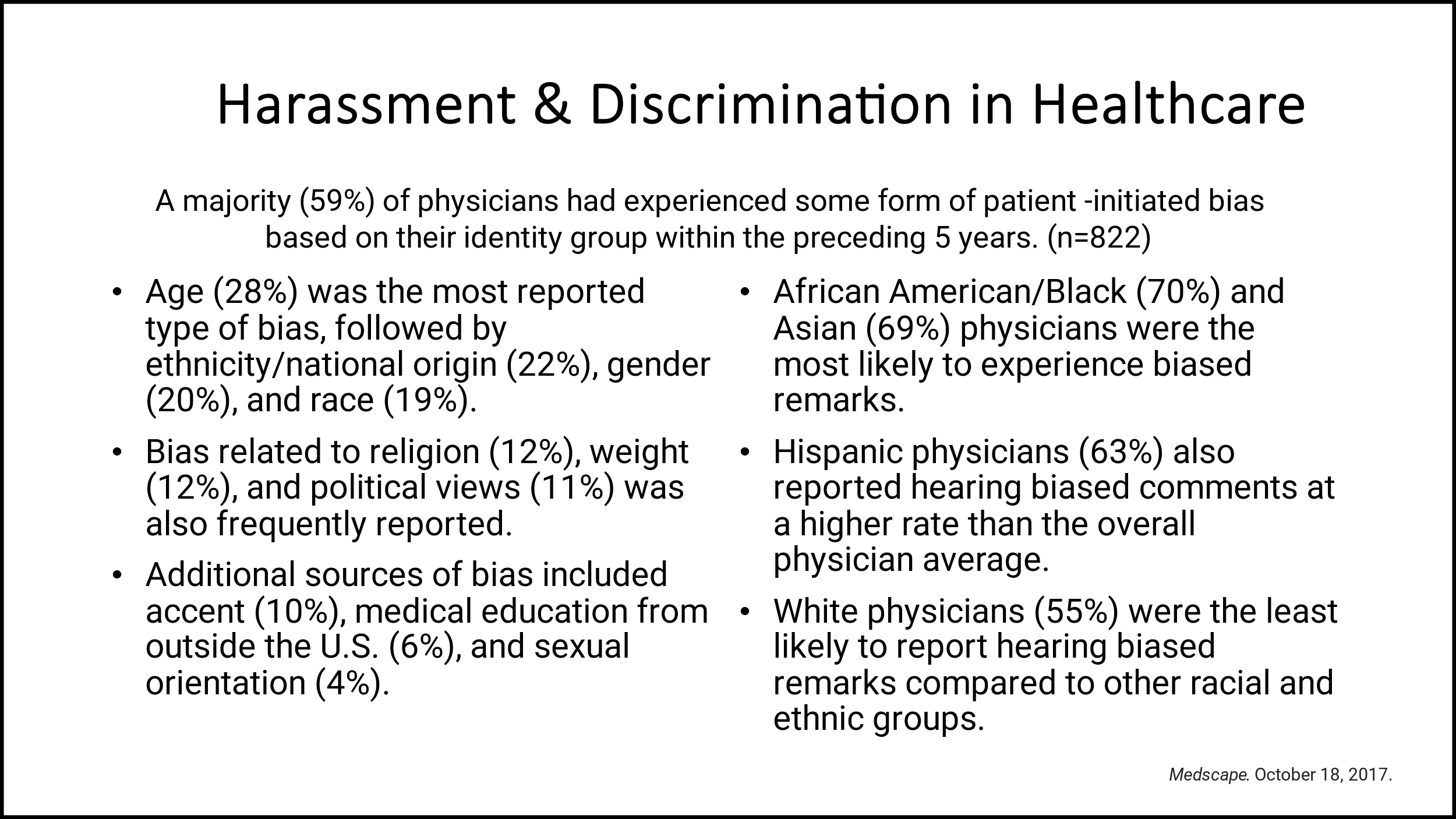


Slide 10: Harassment & Discrimination in Healthcare

*Describe the data as it relates to harassment and discrimination in healthcare.* Data shows that most physicians have experienced some form of patient-initiated bias based on their identity group within the preceding 5 years. The most reported

types of bias are related to a physicians age, ethnicity or national origin, gender, and race. Also, Black and Asian physicians were the most likely racial groups to hear biased comments.

*Cajigal S, et al. Medscape. October 18, 2017.*

**Nearly 50% of residents reported having had experience with at least one form of mistreatment**


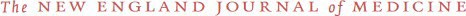

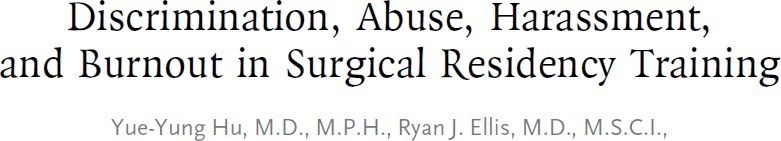

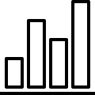

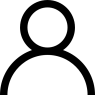

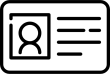


**Mostfrequent source of mistreatment was patients and patient families**

**Residents exposed to discrimination, abuse, or harassment were more likely to have burnout symptoms and suicidal thoughts**

*N Engl J Med*. 2019;381(18):1741-1752.

Slide 11: Article entitled “Discrimination, Abuse, Harassment, and Burnout in Surgical Residency Training

*Share more data on discrimination, abuse, harassment and burnout of trainees (NOTE: this slide can be changed to reflect specialty specific data)::*

This article surveyed 7409 general surgery residents. 60% were male and 40% were female. The

residents exposed to discrimination, abuse, or harassment were more likely to have burnout symptoms. Women were more likely to report burnout symptoms than men *(42.4% vs 35.9%).* Nearly 50% of the residents reported having had experience with at least one form of mistreatment. The most frequent source of mistreatment was from patients and patient families. Overall, residents exposed to discrimination, abuse, or harassment were more likely to have burnout symptoms and suicidal thoughts.

*References:*

*Hu YY, Ellis RJ, Hewitt DB, et al. Discrimination, Abuse, Harassment, and Burnout in Surgical Residency Training. N Engl J Med. 2019;381(18):1741-1752*

Nearly 50% of residents reported Icon: Image made by srip

from www.flaticon.com, retrieved https://[www.flaticon.com/free-icon/bar-](http://www.flaticon.com/free-icon/bar-) chart_900772?term=bar+graph&page=1&position=4&origin=search&related_id=900 772 on March 18, 2023. Image is in public domain and free for personal and commercial purpose with attribution.

Most frequent source of mistreatment Icon: Image made

by Freepik from www.flaticon.com, retrieved https://[www.flaticon.com/free-](http://www.flaticon.com/free-) icon/user_747376?term=person&page=1&position=3&origin=search&related_id=747 376 on March 18, 2023. Image is in public domain and free for personal and commercial purpose with attribution.

Residents exposed to discrimination Icon: Image made by Freepik from www.flaticon.com, retrieved https://[www.flaticon.com/free-](http://www.flaticon.com/free-)

icon/card_1157044?term=name+tag&page=1&position=37&origin=search&related_i d=1157044 on March 18, 2023. Image is in public domain and free for personal and commercial purpose with attribution.

**Harassment of the Medical Trainee**

- Trainees are in uniquely vulnerable positions and are more likely to be targets of patient bias
  - Frequently interface with patients first
  - Limited autonomy in decision making
  - Rotate through rotations frequently with constant turnover with each academic calendar

Slide 12: Harassment of the medical trainee

*Share information about why trainees are vulnerable to patient bias/harassment* As frontline workers, trainees are in uniquely vulnerable positions and are more likely to be targets of patient bias. Some of the reasons behind this is that trainees frequently interface with patient first, they may have limited autonomy in decision making, and go through rotations frequently with constant turnover with each academic year.

*Feel free to add your own potentials reasons for bias/harassment of trainees. References: K. How Should Organizations Support Trainees in the Face of Patient Bias?*

*AMA J Ethics. 2019;21(6):E513-520.*


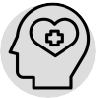

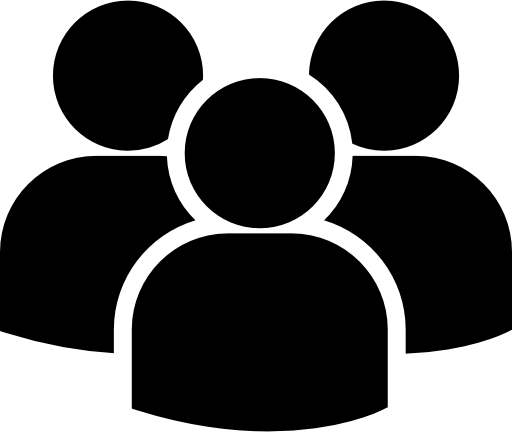

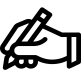

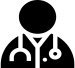


**Impact of Harassment**

**Negatively affects work performance**

**Compromises trainee mental health and learning**

**Long-lasting effects on professional careers**

Slide 13: Impact of Harassment

*Explain the impact on harassment or unwelcome conduct on trainees*

As noted before, harassment or unwelcome conduct, can have a harmful impact. Discrimination toward individuals of minoritized groups undermines efforts to foster inclusion and creates an unwelcoming workplace environment. Unwelcome conduct can have deleterious effect on emotional well-being and attitudes and ultimately, **negatively affects work performance.** For example, it may result in avoidant behaviors, missing work, and neglecting tasks, which impacts patient care.

**Harassment compromises a trainee's well‐being including their mental health and learning.** The emotional or psychological impact can result in trainees developing depression, anxiety, insomnia, and even PTSD. Often individuals with PTSD will develop avoiding behaviors. In this situation, a trainee may start to avoid clinical rotations or work to avoid harassers and there miss critical learning opportunities. Furthermore, burnout can be a result of harassment. Due to burnout, a trainee's ability to focus wanes, engagement with work suffers, andbegins to develop feelings of apathy & hopelessness.

**Harassment is also reported to have long‐lasting effects on professional careers. Individuals share experiencing** lower career satisfaction, lower confidence in clinical abilities and ability to succeed in residency.

*May consider sharing the impact of microaggressions:*

Microaggressions can result in an individual experiencing increased irritability, emotional exhaustion, lack of productivity and poor performance, and feeling out of place and invisible.

##### References:

1. *Willness C. R. SP, Lee K. A meta-analysis of the antecedents and consequences of workplace sexual harassment. Personnel Psychology. 2007;60:127-62.*
2. *Fnais N, Soobiah C, Chen MH, Lillie E, Perrier L, Tashkhandi M, et al. Harassment and discrimination in medical training: a systematic review and meta-analysis. Acad Med. 2014;89(5):817-27.*
3. *Sheehan KH, Sheehan DV, White K, Leibowitz A, Baldwin DC, Jr. A pilot study of medical student 'abuse'.*

*Student perceptions of mistreatmentand misconduct in medical school. JAMA. 1990;263(4):533-7.*

1. *Cabrera MT, Enyedi LB, Ding L, MacDonald SM. Sexual Harassment in Ophthalmology: A Survey Study. Ophthalmology. 2019;126(1):172-4.*

Impact of Harassment Large Icon: Image made

by Freepik from www.flaticon.com, retrieved https://[www.flaticon.com/free-](http://www.flaticon.com/free-) icon/multiple-users- silhouette_33308?term=people&page=1&position=2&origin=search&related_id=333 08 on March 15, 2023. Image is in public domain and free for personal and commercial purpose with attribution.

Negatively affects work performance icon: Image made

by Freepik from www.flaticon.com, retrieved https://[www.flaticon.com/free-](http://www.flaticon.com/free-) icon/writing_1170221?term=hand+writing&page=1&position=1&origin=search&relat ed_id=1170221 on March 22, 2023. Image is in public domain and free for personal and commercial purpose with attribution.

Compromises trainee mental health and learning icon: Image made by photo3idea_studio from [www.flaticon.com,](http://www.flaticon.com/) retrieved https://[www.flaticon.com/free-](http://www.flaticon.com/free-) icon/wellbeing_5980316?term=wellbeing&page=1&position=2&origin=search&relate d_id=5980316 on June 9, 2023. Image is in public domain and free for personal and commercial purpose with attribution.

Long-lasting effects o professional careers icon: Image made by Freepik from www.flaticon.com, retrieved https://[www.flaticon.com/free-icon/doctor_1979863?term=doctor&page=1&position=34&origin=search&related_id](http://www.flaticon.com/free-icon/doctor_1979863?term=doctor&page=1&position=34&origin=search&related_id)

=1979863 on March 22, 2023. Image is in public domain and free for personal and commercial purpose with

attribution.

**What are Barriers to Responding to Patient Harassment?**

Slide 14: What are Barriers to Responding to patient harassment?

*Encourage participants to share their thoughts about what are some barriers to responding to patient harassment. For virtual presentation - Ask participants to add their response to the chat.*

Can you share some barriers to responding to patient harassment that might be directly experienced or witnessed?

*(For virtual)* Please share your response in the chat.

*Beloware some examples of barriers to responding that you can share:*

*-Don’t want to damage patient rapport/ “customer service” model*

*-Concern about retaliation/poor rotation grades*

*-Lack of autonomy or support*

*-Not sure if serious enough to say anything*

*-Don’t know what to say for do*

*-Customer is always right*

*-Worried about getting in trouble with their supervisor*

*-Worried about impacting customer satisfaction (Press Ganey scores, impact annual reviews, and ultimately career)*

*-Was it intentional or not*

*-Believe nothing would happen*

*Barriers to responding when witnessing harassment:*

*Don’t want to embarrass learner*

*-Don’t want to damage patient rapport/ “customer service” model*

*-Concern about retaliation/poor rotation grades*

*-Lack of autonomy or support*

*-Not sure if serious enough to say anything*

*-Don’t know what to say for do*

*-Customer is always right*

*-Worried about getting in trouble with their supervisor*

*-Worried about impacting customer satisfaction (Press Ganey scores, impact annual reviews, and ultimately career)*

*-Was it intentional or not*

*-Believenothing would happen*

*-Too busy*

*-Don’t know how to file*

**Tools for Responding to Patient-Initiated Harassment**

Slide 15: Tools for Responding to Patient-Initiated Harassment

*This slide is intended as a transition slide to the next part of the workshop where participants will be able to obtain skills that will allow them to respond to patient- initiated harassment*

Next, we will transition to the next part of our workshop where you will be able to obtain the skills needed to respond to patient-initiated harassment.

# Audience Poll

**Add QR Code Here**

1. Open your Camera App.
2. Aim your camera at the QR code and make sure it is in focus.
3. Your QR code should automatically be scanned.
4. Tap on the pop-up text that appears.
5. You will be brought to the QR code link/information.

Slide 16: Audience Poll #2

*In order to assess previous communication training on responding to harassment and comfort level, the following poll questions below should be answered by the participants. You can create a poll through Microsoft forms, google forms, etcs.*

*Would recommend adding a QR code to the slide so that participants are able to quickly access the survey.*

*Poll Question and Answer options:*

*#1: Have you previously received training on techniques for responding to patient harassment?*

- *Yes*
- *No*

*#2: If you experience harassment or observe harassment of a colleague. How prepared do you feel to respond to the harassment?*

- *Very prepared*
- *Slightly prepared*
- *Neutral*

*Slightly unprepared*

- *Very unprepared*

*Read the following to the participants:*

We have a couple more poll questions we would like you to answer. Have you previously received training on techniques for responding to patient harassment? Also, if you experience harassment or observe harassment of a colleague. How prepared do you feel to respond to the harassment?

Again, please open the camera app on your phone and aim the camera at the QR code. The QR code should be automatically scanned. Tap on the pop up text that appears on your screen. You will then be brought to the survey link for Audience Poll #2. Please fill out this survey.

*You can create a Results link on your slide that can take participant to a website with the poll answers. Review the poll results with the participants.*

**Current harassment training is insufficient**

- Often passive or online
- Does not teach communication skills for responding to harassment
- Does not teach skills for supporting trainees

Slide 17: Current harassment training is insufficient

*Review the lack of formal patient-initiated harassment training.*

Currently most harassment training is insufficient. Sexual harassment training is the most common training offered. However, this often does not address other identity-based harassment. Furthermore, the sexual harassment training often does not address harassment that come from

patients. Furthermore, the trainings are more passive or online. Trainings often do not teach communications skills for responding or ways of supporting trainees or others on our care teams.

*Scruggs BA, Hock LE, Cabrera MT, Wang K, Oetting TA, Abramoff MD, Shriver EM. A*

*U.S. Survey of Sexual Harassment in Ophthalmology Training Using a Novel Standardized Scale. Journal of Academic Ophthalmology. 2020;12(01):e27-e35.*


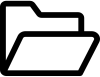

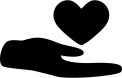

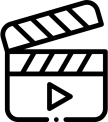


**General considerations**

**Assess clinical situation**

**Responses should Be prepared and aim for empathy practice responses**

Slide 18: General Considerations

*Review general considerations that may be needed prior to responding to harassment:*

It is important that you that you take several considerations into account prior to responding to harassment.

First, assess the situation. It may not be appropriate to respond in certain situations such as in an

emergency or if a patient is clinically unstable. Also assess if a patient is under the influence of substances such as waking from general anesthesia or if there is a personal safety concern. It is also important to assess a patient’s current mental state. Take into consideration a patient’s baseline or altered mental capacity. For example, if a patient is delirious, manic or psychotic. In some situations, it may not be appropriate to respond to the patient, but IT IS always important to support team members.

Other considerations include either effective responses are most often active, positive, and polite. Responses should aim for empathy and address inappropriate behavior in a nonjudgmental way. Consider your goals to stop the behavior and educate the patient. Conversations can be both corrective

and respectful. Cultivation of a therapeutic alliance—establishing rapport, exploring reasons for bias, expressing empathy, and focusing on health concerns. Identifying the behavior as discriminatory or harassing and recognizing that it may be motivated by fear, anxiety, loss of control, or past experiences

Physicians should **E**xpect that mistreatment will happen. Expecting mistreatment will happen helps faculty prepare for future episodes and develop strategies to address concerns. Part of being prepared is to **r**ecognize when mistreatment, unwelcome conduct, or harassment occurs. Some instances are obvious where others are more subtle. Some physicians become desensitized to these experiences. If mistreatment targets a trainee of a different demographic than the supervising physician, they are less likely to recognize the incidence

It is important to recognize that unwelcome conduct is emotionally charged and often come out of nowhere. Therefore, we must not only be prepared to recognize it but also be ready with a response when it is most effective at the time of harassment. This takes practice, just like breaking bad news to a patient. You are not expected to memorize all possible responses but consider picking one or two that feel natural to you and that would work in a number of scenarios.

*References:*

1. *Goodman DJ. Responding to biased or offensive comments. Promoting Diversity and Social Justice: Educating People from Privileged Groups. New York: Routledge; 2011. Eisenberg EH, Kieffer KA. Use of Simulated Patient Encounters to Teach Residents to Respond to Patients Who Discriminate Against Health Care Workers. J Gen Intern Med. 2019;34(5):764-768. doi:10.1007/s11606-019-04881-3*
2. *Goldenberg MN, Cyrus KD, Wilkins KM. ERASE: a New Framework for Faculty to Manage Patient Mistreatment of Trainees. Acad Psychiatry. 2019;43(4):396-399. doi:10.1007/s40596-018-1011-6*

Assess clinical situation icon: Image made

by Freepik from www.flaticon.com, retrieved https://[www.flaticon.com/free-](http://www.flaticon.com/free-) icon/folder_1383970?term=file+folder&page=1&position=5&origin=search&related_i d=1383970 on March 22, 2023. Image is in public domain and free for personal and commercial purpose with attribution.

Responses should aim for empathy icon: Image made

by Freepik from www.flaticon.com, retrieved https://[www.flaticon.com/free-](http://www.flaticon.com/free-) icon/hand-with- heart_67468?term=hand++with+heart&page=1&position=1&origin=search&related_i d=67468 on March 15, 2023. Image is in public domain and free for personal and commercial purpose with attribution.

Be prepared and practice responses icon: Image made

by Freepik from www.flaticon.com, retrieved https://[www.flaticon.com/free-](http://www.flaticon.com/free-) icon/video_1179120?term=movie&page=1&position=1&origin=search&related_id=1 179120 on March 22, 2023. Image is in public domain and free for personal and commercial purpose with attribution.


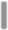

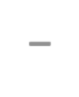

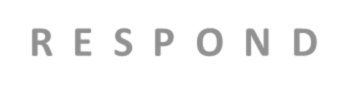


**Response Toolkit**

**I – R E S P O N D**

https://eyerounds.org/tutorials/sexual-harassment-toolkit/index.htm

Slide 19: Response Toolkit

*For a live presentation, if participants were not given the handouts at the beginning of the workshop, handout the double sided* ***I‐RESPOND Toolkit for Addressing Patient‐Initiated Identity‐Based Harassment*** *handout to the participants now. For a virtual presentation, the* ***I‐ RESPOND Toolkit for Addressing Patient‐Initiated Identity‐ Based Harassment (Handout)*** *should either be sent electronically to participants prior to the workshop or sent as a file though the chat.*

Now we will review our **I‐RESPOND Toolkit for Addressing Patient‐Initiated Identity‐Based Harassment**. These communication strategies may be used to respond to multiple forms of identity- based harassment initiated by patients in the clinical setting.

References:

1. *Goodman DJ. Responding to biased or offensive comments. Promoting Diversity and Social Justice: Educating People from Privileged Groups. New York: Routledge; 2011.*
2. *Hock LE, Scruggs B, Oetting TA, Abramoff MD, Shriver EM. Tools for Responding to Patient- Initiated Verbal Sexual Harassment. https://EyeRounds.org/tutorials/sexual-harassment- toolkit/index.htm. Updated Posted August 20, 2019. Accessed April 8, 2020.*
3. *Goldenberg MN, Cyrus KD, Wilkins KM. ERASE: a New Framework for Faculty to Manage Patient Mistreatment of Trainees. Acad Psychiatry. 2018.*
4. *Wheeler DJ, Zapata J, Davis D, Chou C. Twelve tips for responding to microaggressions and overt discrimination: When the patient offends the learner. Med Teach. 2019;41(10):1112 -1117. doi:10.1080/0142159X.2018.1506097*


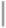

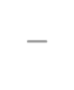

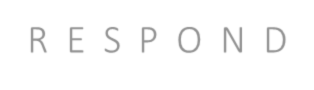


I – R E S P O N D

Use “I” Statements

*“I feel uncomfortable when you comment on my physical appearance/race/age/religion/etc.”*

Slide 20: Use “I” Statements

*Discuss the use of “I” statements as an approach to respond:*

In some situations, it may be effective to express how the person’s unwelcome conduct made you feel. For example, you can share: “I feel upset when I hear comments like that because it makes me feel like I don’t belong here.” Another example is: “When I hear comments like that, it makes me feel

like you think I am only here because I am a minority, not because I can do the work.”


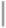

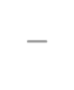

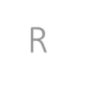

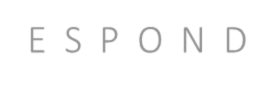


I – R E S P O N D

Repeat and Clarify Statement

*“Help me understand what you mean by that.” or “I heard you say . Will you clarify what you meant?”*

Slide 21: Repeat and Clarify Statement

*Discuss the approach of repeating and clarifying your statements.*

It can be also very effective to have the individual who made an unwelcome statement repeat or clarify what they said. An example includes stating: “I think I heard you say . What makes you believe that?” or What do you mean by that?” or “I don’t understand what you meant when you said . Can you elaborate?”


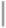

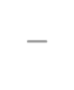

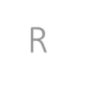

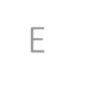

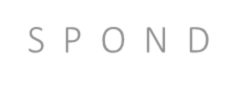


I – R E S P O N D

Emphasize Shared Goals

*“I want to give you the best care that I can, but comments like that distract me and reduce my ability to focus on your care. Let’s keep the conversation professional”*

Slide 22: Emphasize Shared Goals

*Discuss the approach of emphasizing shared goals.*

Emphasizing shared goals of care can be another way of responding. You can respond by saying: “I want to give you the best care that I can, but your comments distract me and reduce my ability to care for you to the best of my ability” or “I want to provide the best care that I can, but your comments make me feel [xyz] and prevent me from caring for you to the best of my ability.”

Another example is “It’s difficult for me to focus on your health when you make comments like that” or “We are here to focus on your health.”


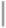

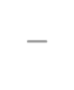

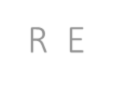

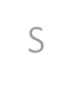

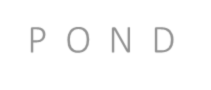


I – R E S P O N D

Set Boundaries

*“Our hospital policy does not allow for discrimination on the basis of race/religion/gender/sexual orientation.*

*If you continue, I will have to leave the*

*room.”*

Slide 23: Set Boundaries

*Discuss the approach of setting boundaries*

Depending on the degree of harassment, setting boundaries can be very important in situations where patient-initiated harassment has occurred, especially if you are in an unsafe environment. Some potential responses include: “I’m leaving the room because I don’t feel comfortable with your behavior.” Another example is: “At this hospital/clinic, we treat each other with respect. We cannot permit/tolerate that kind of language/behavior” or “I don’t tolerate racist/sexist/homophobic jokes in my workspace. If you continue to comment on my physical appearance/race/age/religion/etc., I will have to leave the room.”


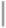

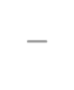

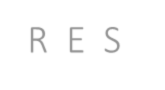

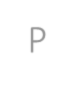

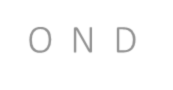


I – R E S P O N D

Patient Actions rather than Person

*“I felt disrespected when you said that,” is less likely to make a harasser respond defensively than, “You are disrespectful.”*

Slide 24: Patient Actions rather than Person

*Discussion the importance of focusing on a patient’s actions rather than the person.*

When responding, it is important to focus on the actions and not on the individual who made the unwelcome conduct. For example, try to avoid judgement and name calling. Consider responding with "your behavior or comment could be interpreted as a sexist/homophobic/racist remark” or your comment “generalizes an entire [group] in a discriminatory way” or “I’m not sure what my background has to do with the care I can provide.”


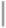

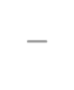

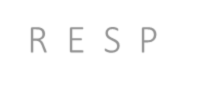

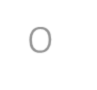

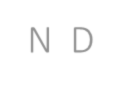


I – R E S P O N D

Offer an Alternative

*“I would prefer if you call me, ‘Doctor’, rather than ‘baby’ or ‘honey’.”*

Slide 25: Offer and Alternative

*Discuss responses that “offer” and alternative to an unwelcome comment.* Another response to consider is to offer the person who made the unwelcome comment an alternative. For example, you could respond by

saying “I would prefer if you call me, ‘Doctor’, rather than ‘baby’ or ‘honey.’ “


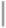

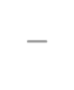

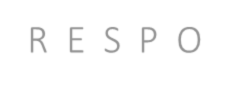

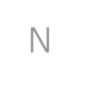

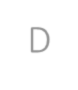


I – R E S P O N D

Separate INtent from Impact

*“I’m sure you didn’t mean to be hurtful when you said that, but it made me feel…”*

Slide 26: Separate Intent from Impact

*Discuss the approach of separating one’s intent from the impact.*

Sometimes patients are not meaning to be hurtful. Therefore, it is important to respond by focusing on the impact of what was said versus the patient’s potential intent. Examples of how you could respond include: “You may mean well, but we are more concerned with our resident’s skills and abilities than the way they look” or “Maybe you were just trying to be funny, but I found that joke offensive because

. ” Another example is “Though I’m sure you

weren’t intentionally trying to be hurtful, I felt when you said ” or “Statements like that make me think that you feel that I’m only here because of my background, not because of my abilities” or “I wonder how an individual from [specific group] would feel if they heard a comment like that.”


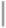

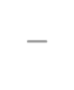

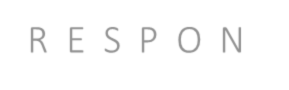

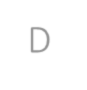


I – R E S P O N D

Don’t Use Humor

*Use humor with caution Avoid laughing*

Slide 27: Don’t Use Humor

*Discuss the importance of avoiding humor.*

It is important to use humor with caution as exaggeration of an inappropriate comment or gentle sarcasm may be misconstrued as reinforcement or encouragement of the unwelcome conduct. Also, when you are witnessing the harassment laughing can cause isolation to the person who is experiencing the harassment.

**Practice Skills**

Slide 28: Practice Skills

*Facilitator should explain that we will now begin to practice skills.*

Now we will have an ability to practice the response skills we just learned.

Video Scenario 1:

**Insert Scripted Video Scenario 1 "Inappropriate / No Response" Clip Here**

Slide 29: Scenario 1

*Play the video for participants*

Now we will show a video scenario showcasing a patient scenario. Afterwards you will have an opportunity to practice how you would respond in small break out groups.

**How would you respond?**

**4-minute small group discussion**

Slide 30: How would you respond? – Scenario 1

*Facilitator should explain to participants that they will break into groups of 2-3 for 4-5 minutes to role play their responses to the patient scenario. Facilitator should emphasize that each participant should rehearse how they would respond to the inappropriate patient comment(s) in their own words. Pairs of participants may then discuss which responses may be effective or ineffective.*

***For virtual presentations*** *– A breakout room will need to be set up with 2-3 participants for 4-5 minutes. Make sure the Practice Skills Small Group Handout sent as a file through the chat.*

***For live presentations*** *- It is easiest if participant’s role play with someone physically nearest them. The Practice Skills Small Group Handout should be passed out to participants.*

We will break into groups of 2-3 for 4 minutes to role play your response to a patient scenario. Practice how you would respond if you were the resident/student. It is important that each of you rehearse how you would respond to the inappropriate patient comment in your own words. Refer to the “I-RESPONDToolkit. Also, consider discussing which responses may be effective or ineffective in this scenario. Please refer to the “Practice Skills Small Group Handout” for the script and further instructions.

*After 4-5 minutes, the facilitator should reconvene entiregroup and ask for 2-3 pairs to share responses that seemed effective and responses that may be ineffective, including those that did not work well from experience. Facilitator may share their own response script to encourage discussion. The large group discussion may last about 2-3 minutes.*

Please share what you discussed in your small groups. Share some responses that seems effective and responses that may be ineffective.

*Potential scripts:*

It makes me uncomfortable when you comment about my appearance.

Let’s keep our conversation professional so I can focus on providing you with the best care

“I would prefer that we keep the conversation about your health and

comments like that distract from my ability to offer you the best care.” “I’m sure you didn’t mean to be hurtful, but I feel uncomfortable when you comment on my appearance.

“I want to give you the best care that I can so let’s keep our conversation professional”.

Putting it all together

**Insert Scripted Video Scenario 1 "Bystander Correct Response with Debrief" Clip Here**

##### Slide 31: Scenario 1 (Video 2) Putting it all together

*Participants will watch video where a trainee is being verbally harassed by a patient and the trainee responds appropriately.*

Let's watch this video again with a different response from the trainee.


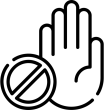

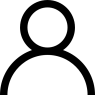

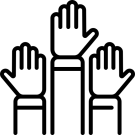


What if you witness harassment?

**Silent collusion**

Being complicit by staying silent

**Bystander**

A person who is present but not involved

speaks or acts in

**Upstander**

A person who

support of an

attacked or bullied

individual being

Slide 32: What if you witness harassment?

*Transition to discuss witnessing harassment versus directly experiencing*

*harassment. Define the termssilent collusion, bystander, and upstander.* Shortly we will look at a scenario that includes witnessing harassment. However, let's first define a couple terms. Silent collusion involves being complicit by staying silent. Bystander is a person who is present but not involved. On the otherhand, an upstander is a person who speaks or acts in support of an individual being attacked or bullied.

Silent collusion icon: Image made

by Freepik from www.flaticon.com, retrieved https://[www.flaticon.com/free-](http://www.flaticon.com/free-) icon/banned_4144469?term=stop&page=1&position=3&origin=search&related_id=4 144469 on March 22, 2023. Image is in public domain and free for personal and commercial purpose with attribution.

Bystander Icon: Image made

by Freepik from www.flaticon.com, retrieved https://[www.flaticon.com/free-](http://www.flaticon.com/free-) icon/user_747376?term=person&page=1&position=3&origin=search&related_id=747 376 on March 18, 2023. Image is in public domain and free for personal and commercial purpose with attribution.

Upstander Icon: Image made

by Freepik from www.flaticon.com, retrieved https://[www.flaticon.com/free-](http://www.flaticon.com/free-) icon/raise- hand_2634173?term=raise+hand&page=1&position=10&origin=search&related_id=2 634173 on March 22, 2023. Image is in public domain and free for personal and commercial purpose with attribution.


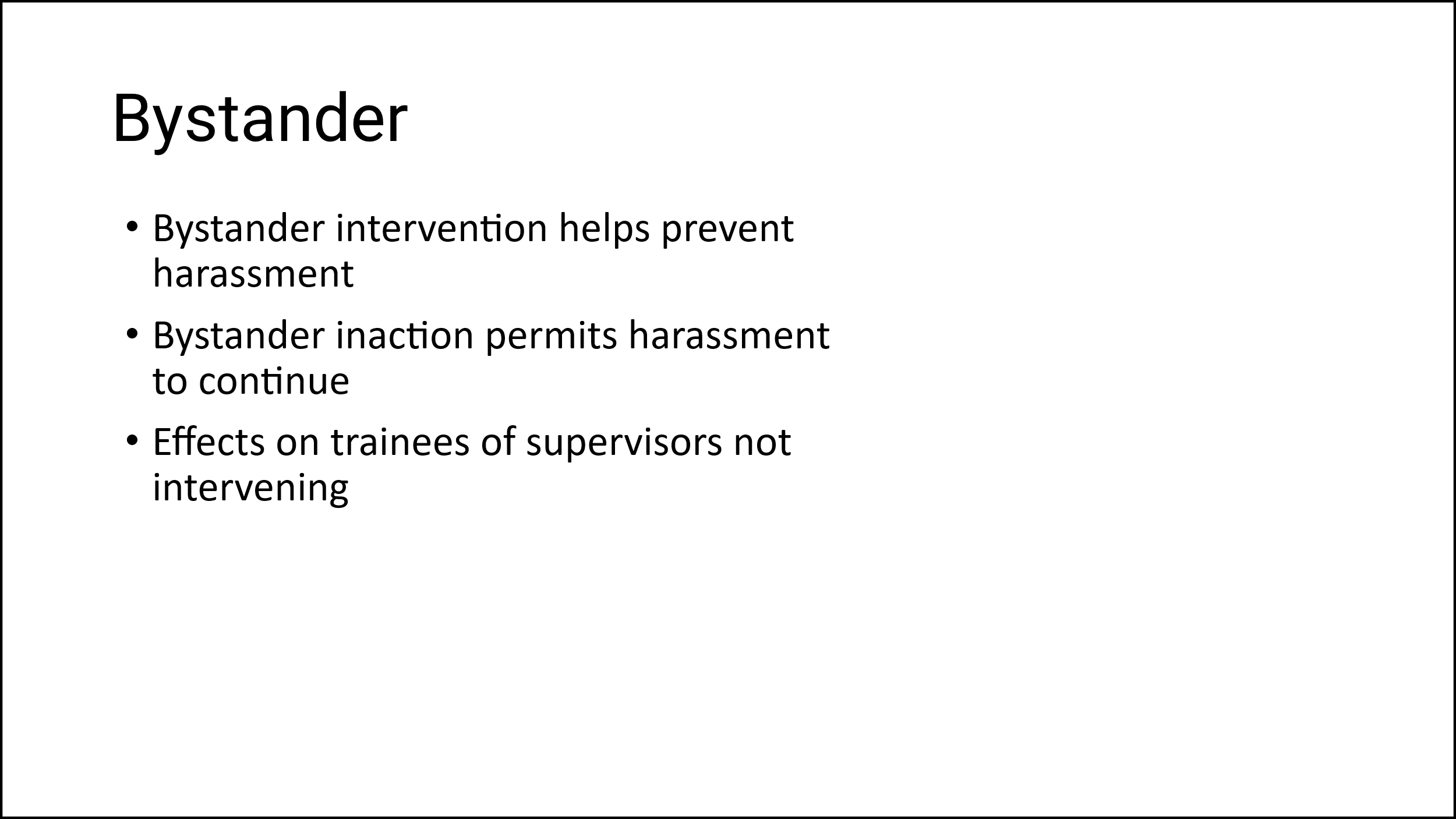


Slide 33: Bystander

*Explain what a bystander is*

When a bystander intervenes, it helps prevent harassment. However, inaction permits harassment to continue. In situations of harassment towards trainees, a supervisor not intervening can have negative effects on trainees


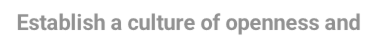

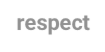

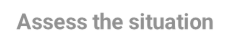

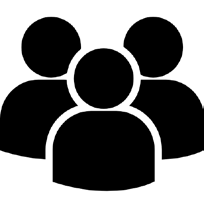

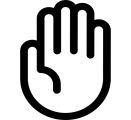


If You Observe Harassment of Trainee or Colleague……

**Establish a culture of openness and**

**respect**

**Assess the situation**

Slide 34: If you Observe Harassment of Trainee or Colleague (slide 1)

*Discuss what should be done to address situations where a participant might observe harassment of a trainee or colleague. For live presentations, participants should have already received the* ***I‐RESPOND Toolkit for Addressing Patient‐Initiated Identity‐ Based Harassment*** *handout. For virtual presentations, the* ***I‐RESPOND Toolkit for Addressing Patient‐Initiated Identity‐Based Harassment (Handout 2)*** *should either be sent electronically to participants prior to the workshop or sent as an attachment though the chat.*

*(for a live presentation)* For the next two slides please refer to the back of your I-RESPOND Toolkit.

*(for a virtual presentation)* For the next two sides please open the ***I‐RESPOND Toolkit for Addressing Patient‐Initiated Identity‐Based Harassment (Handout 2)***

It is important to establish a culture of openness and respect before an incident of harassment occurs. Expect that harassment will happen and recognize when it occurs. One way to start the discussion is to say: “I wish that inappropriate comments and harassment by patients and visitors did not occur. But it does. I want to hear when things like this happen. It’s important that everyone feel safe and supported here.”

It is also important to assess the situation. Does the person who was harassed appear uncomfortable or upset? Nonverbal cues should clue you in to whether the person desires help handling the situation.

Establish a culture of openness and respect Icon: Image made

by Freepik from www.flaticon.com, retrieved https://[www.flaticon.com/free-](http://www.flaticon.com/free-) icon/multiple-users- silhouette_33308?term=people&page=1&position=2&origin=search&related_id=333 08 on March 15, 2023. Image is in public domain and free for personal and commercial purpose with attribution.

Assess the situation Icon: Image made

by Freepik from www.flaticon.com, retrieved https://[www.flaticon.com/free-](http://www.flaticon.com/free-) icon/hand_3898664?term=stop+hand&page=1&position=4&origin=search&related_i d=3898664 on March 15, 2023. Image is in public domain and free for personal and commercial purpose with attribution.


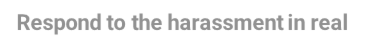

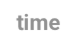

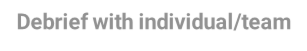

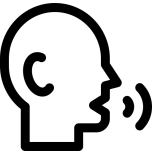

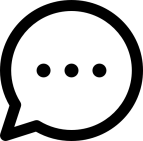


If You Observe Harassment of Trainee or Colleague……

**Respond to the harassment in real**

**time**

**Debrief with individual/team**

##### Slide 35: If you observe harassment of trainee or colleague (slide 2)

*Continue to discuss ways to respond if a participant observes harassment of a trainee or colleague.*

It is critical that you respond to the harassment in real time. Regarding the previous scenario, imagine

the attending or supervising physician was present. As an upstander, the supervisor could respond by saying the following: “Dr. Y is a skilled physician and a talented surgeon. That’s far more important than his/her [appearance/identity/background]/Most of our physicians prefer to be called, ‘doctor" or “Mr. Z, we want to give you the best care we can and ask that you treat all of our team members with respect.” Another option is to say: “We don’t tolerate that kind of language here/Let’s keep it professional.” You can also consider redirecting the patient to prevent further harm. It is also important to provide the individual that is harassed with an opportunity to leave the room.

Finally, it is important to debrief with the individual who was harassed and the team regarding the harassment. During the debrief with the individual who was harassed you could say: “That was a tough encounter. How are you doing?”. “I would like to take some time to acknowledge and reflect on how that encounter felt for everyone" or “How do you think the encounter went? How can I/we address the situation differently next time to ensure a better outcome for everyone involved?”

Make it your own:

Respond to the Harassment Icon: Image made by Freepik from www.flaticon.com, retrieved https://[www.flaticon.com/free-](http://www.flaticon.com/free-) icon/rumor_4287352?term=speak&page=1&position=5&origin=search&related_id=4 287352 on March 22, 2023. Image is in public domain and free for personal and commercial purpose with attribution.

Debrief with individual/team Icon: Image madeby Freepik from www.flaticon.com, retrieved https://[www.flaticon.com/free-](http://www.flaticon.com/free-) icon/messenger_1370907?term=chat&page=1&position=2&origin=search&related_i d=1370907 on March 22, 2023. Image is in public domain and free for personal and commercial purpose with attribution.

Scenario 2

**Insert Scripted Video Scenario 2 "Bystander Responds Out of Turn (Incorrect Response)" Clip Here**

Slide 36: Scenario 2

*Participants will watch video where a trainee is being verbally harassed by a patient and the supervising physician responds.*

Let's watch our next scenario.

**What went wrong in this scenario?**

**How would you respond differently to the patient?**

**How would you support the learner?**

**4-minute small group discussion**

##### Slide 37: How would you respond? – Scenario 2

*Participants will have an opportunity to answer the following questions on the slide in small groups. Patient will break up into groups of 2-3 for 4 min to discuss the video.*

*For virtual presentations –A breakout room will need to be set up with 2-3 participants for 4 minutes.*

You will now have an opportunity to reflect on the video you just saw. Think about what went wrong in

this scenario, how you might respond differently to the patient, how you would support the learner, and how you would respond to the learner. You will break into [the same] groups of 2-3 for 4 min. The questions on this slide are included on your “Practice Skills Small Group Handout.” As you are answering these questions, please refer to your

I-RESPOND toolkit.

*After 4 minutes, the facilitatorshould reconvene the entire group and ask for 2-3 pairs to share responses. Facilitator may share their own responses to encourage discussion. The large group discussion may last about 5 minutes.*

Please share what you discussed in your small groups.

*Potential questions to ask participants:*

*What type of mistreatment do you* ***recognize****? What went* ***wrong*** *in this scenario?*

*How might the faculty memberin the case* ***address*** *the situation in real time? (suggest specific language)*

*How might faculty* ***support*** *the learner?*

*What institutional interventions might be needed to* ***establish/encourage*** *a positive culture?*

**Wrap-Up**

Slide 38: Wrap-Up

*Transition to the wrap-up portion of the workshop*

We will transition to the wrap-up portion of this workshop.

### Graduate Medical Education Guidelines


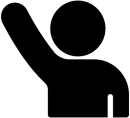

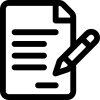

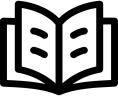


#### Reporting

Contact immediate supervisors

#### Documentation

Document pertinent details, relevant quotes and facts about the encounter in the EMR

#### Resources

Disruptive Patient and Visitor Program Office of Sexual Misconduct Response Office of Equal Opportunity and Diversity

Slide 39: Graduate Medical Education Guidelines

*Share the GME guidelines for harassment.*

It is important that you encourage reporting and documentation of patient harassment.

The GME guidelines for reporting include contacting your supervisor immediately. Report harassment that threatens safety or creates an intimidating, hostile, or offensive work environment.

Riskonnect Patient Safety Incident Reporting System

Document incidents of harassment in the Electronic Medical Record. Include the pertinent details, relevant quotes, and facts about the encounter.

You can find the UI Healthcare GME guidelines on responding to harassment on MedHub.

*Examples of appropriate documentation include:*

- *I introduced myself to the patient and he responded “Where the hell are YOU from?’ Send the American staff to see me.” I attempted to explain my role, but he talked over*

*me; referencing the color of myskin; persisted using insulting, racist putdowns.*

- *Patient shouted at me: “You are a f ing idiot !” Patient leaned forward in a hostile manner; pointed his finger towards my face.*
- *You are one good looking little lady. (Makes gestures with his hands outlining her breasts.) When I was your age we would really make it in the sack. I bet you would be awesome. Have you ever done a guy my age? I can teach you a thing or two.*
- *I entered the room, explain the procedure and the patient responded: “I thought you had to be older than 10 to work here. Must everybody here be gay?!!! I’m for diversity but this is ridiculous. And while you’re at it go back to school. Learn on somebody else.”*
- *“I’m not opposed to you Chinese being in our country, but I just can understand you. Get somebody who knows how to speak f English. If you can’t speakour language go back to where you came from.”*
- *When I referenced previous recommendations attempting to verify for compliance patient responded: “What are you a retard? Or I guess I’m supposed to say ID? Take that thing off your head and maybe you’ll hear better. I’ve said a million times I’m not going to do that. Let me say it slowly – no retard ID idiot.”*
- *Several resources are available:*
- *(Insert GME Resources at institution)*

Reporting Icon: Image made by Flat Icons Design from www.flaticon.com, retrieved https://[www.flaticon.com/free-](http://www.flaticon.com/free-) icon/ask_8703211?term=raising+hand&page=1&position=7&origin=search&related_i d=8703211 on March 24, 2023. Image is in public domain and free for personal and commercial purpose with attribution.

Documentation Icon: Image made by Freepik from www.flaticon.com, retrieved https://[www.flaticon.com/free-](http://www.flaticon.com/free-) icon/contract_684831?term=pen+and+paper&page=1&position=1&origin=search&re lated_id=684831 on March 27, 2023. Image is in public domain and free for personal and commercial purpose with attribution.

Resources Icon: Image made by Freepik from www.flaticon.com, retrieved https://[www.flaticon.com/free-](http://www.flaticon.com/free-) icon/book_2224395?term=books&page=1&position=51&origin=search&related_id=2 224395 on March 27, 2023. Image is in public domain and free for personal and commercial purpose with attribution.

Key points

- Expect for harassment and mistreatment by patients and their families to occur
- Recognize what is happening and have a scripted response prepared
- Aim for empathy and address inappropriate behavior in a nonjudgmental way
- Understand that the organization support you and is committed to providing a safe and hospitable workplace

Slide 40: Key Points

*Review keytake away points*

Here are a few key take away points from the workshop today:

First, expect for harassment and mby patients and their families tooccur. Expecting mistreatment will happen helps you to prepare for future episodes and develop strategies to address concerns **Also, r**ecognize when mistreatment occurs. Some instances of mistreatment are obvious; others are subtle. It is important to not only recognize mistreatment, unwelcome conduct or harassment and but also have

a scripted response prepared. Aim for empathy and address inappropriate behavior in a nonjudgmental way. Finally, understand that the organization support you and is committed to providing a safe and hospitable workplace.

References:

Hock LE, Barlow PB, Scruggs BA, Oetting TA, Martinez DA, Abramoff MD, Shriver EM. Tools for Responding to Patient-Initiated Verbal Sexual Harassment: A Workshop for Trainees and Faculty. *MedEdPORTAL.*

2021;17:11096.


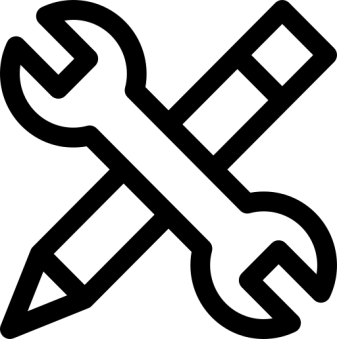


Take Home Point

What is one skill from this workshop you want to remember?

Slide 41: What is one skill from this workshop you want to remember?

*Review skills that participants gained from this workshop. Have participants share their skill (in the chat for virtual presentations).*

Please share one skill from this workshop you want to remember.

Large Icon: Image made by Flat Icons Design from www.flaticon.com, retrieved https://[www.flaticon.com/free-](http://www.flaticon.com/free-) icon/skills_1979383?term=skill&page=1&position=20&origin=search&related_id=197 9383 on March 27, 2023. Image is in public domain and free for personal and commercial purpose with attribution.

***“We are in this together. If we all speak up against identity-based harassment, we can transform the culture in our institution and in medicine.”***

Slide 42: We Stand Together

*End the presentation with the quote on this slide. Read the quote to the participants.*

Responding to Identity-Based Patient-Initiated Discrimination & Harassment

Questions?

Slide 43: Questions

Responding to Identity-Based Patient-Initiated Discrimination & Harassment

**Thank You**

Slide 44: Thank you

Responding to Identity-Based Patient-Initiated Discrimination & Harassment

**Additional Content**

Slide 45: Additional Content

*This is information that can be shared during the presentation if a question comes up or if time permits.*


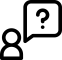


Discriminatory Patient Preference Requests

- “Help me understand your request.”
- “All team members are very qualified. Our top priority is that you receive the best care, and I know that our team members can provide that.”
- “We want to provide you with excellent care and believe that

is the right person to do so.”

AMA J Ethics. 2019;21(6):E521-529

**Scripted Responses to Patient Preference Requests**

Slide 46: Discriminatory Patient Preference Requests

*Review responses to patient preference requests due to a discriminatory reason.*

Patient can have various reasons to request a different provider. However, when a request is made due to discriminatory reasons, the following are scripted responses that you can use:

- “Help me understand your request.”
- “All team members are very qualified. Our top priority is that you receive the best care, and I know that our team members

can provide that.”

- “We want to provide you with excellent care and believe that is the right person to do so.”

Scripted Responses to Patient Preference Requests Icon: Image made

by Freepik from www.flaticon.com, retrieved https://[www.flaticon.com/free-](http://www.flaticon.com/free-) icon/request_1436708?term=request&page=1&position=6&origin=search&related

_id=1436708 on June 9, 2023. Image is inpublic domain and free for personal and commercial purpose with attribution.
