# Supplementary material for: An Innovative Curriculum to Empower Trainees and Faculty to Address Patient-Initiated Identity-Based Misconduct in the Clinical Learning Environment
Source: MedEdPORTAL. 2026 Apr 9;22:11591. doi: 10.15766/mep_2374-8265.11591 (PMC13061878; doi:10.15766/mep_2374-8265.11591)
Supplement: Supplementary file 1 — I-RESPOND Toolkit.docxFacilitator Guide.docxEvaluations.docxPresentation.pptxScenario Scripts.docx [file mep_2374-8265.11591-s001.zip › C. Evaluations.docx]

This appendix provides the evaluation instruments used in the I-RESPOND curriculum study. These materials include the post facilitator training evaluation, retrospective pre–post survey administered immediately after the workshop, as well as the follow-up survey distributed 6–14 weeks later. The surveys are designed to assess participant confidence in addressing patient-initiated misconduct, likelihood of applying I-RESPOND strategies, and reported use of those strategies in clinical practice. The retrospective pre–post survey should be administered once at the end of each workshop session, while the follow-up survey should be distributed electronically to all participants several weeks after completion. These instruments are intended for program evaluators and faculty facilitators who wish to replicate or adapt the study design for their own institutional settings.

**Part One: Post Facilitator Training Evaluation**

| After completing today’s training, how prepared do you feel to: | | | | | | |
| --- | --- | --- | --- | --- | --- | --- |
|  | | **Not Prepared** | **Minimally Prepared** | **Moderately Prepared** | **Very Prepared** | **Extremely Prepared** |
| **1** | Introduce the concept of Patient-Initiated Identity-Based Harassment to learners? | **⃝** | **⃝** | **⃝** | **⃝** | **⃝** |
| **2** | Explain the relevant background research/evidence for Patient-Initiated Identity-Based Harassment to learners? | **⃝** | **⃝** | **⃝** | **⃝** | **⃝** |
| **3** | Discuss how to effectively respond to Patient-Initiated Identity-Based Harassment using the I-RESPOND toolkit? | **⃝** | **⃝** | **⃝** | **⃝** | **⃝** |
| **4** | Facilitate the role-playing activities involved with the workshop? | **⃝** | **⃝** | **⃝** | **⃝** | **⃝** |
| **5** | Manage the group discussion components of workshop? | **⃝** | **⃝** | **⃝** | **⃝** | **⃝** |
| **6** | Use the necessary technology involved for the workshop? | **⃝** | **⃝** | **⃝** | **⃝** | **⃝** |

| After completing today’s training, how prepared do you feel to: | | | | | | |
| --- | --- | --- | --- | --- | --- | --- |
|  | | **Not Prepared** | **Minimally Prepared** | **Moderately Prepared** | **Very Prepared** | **Extremely Prepared** |
| **7** | Deliver the DIDACTIC portion of the workshop accurately? | **⃝** | **⃝** | **⃝** | **⃝** | **⃝** |
| **8** | Facilitate the ROLE-PLAYING portion of the workshop accurately? | **⃝** | **⃝** | **⃝** | **⃝** | **⃝** |
| **9** | Facilitate the DISCUSSION portion of the workshop accurately? | **⃝** | **⃝** | **⃝** | **⃝** | **⃝** |
| **10** | Facilitate the full workshop within the expected time? | **⃝** | **⃝** | **⃝** | **⃝** | **⃝** |

| After completing today’s training, how prepared do you feel to: | | | | | | |
| --- | --- | --- | --- | --- | --- | --- |
|  | | **Not Prepared** | **Minimally Prepared** | **Moderately Prepared** | **Very Prepared** | **Extremely Prepared** |
| **11** | Manage difficult conversations such as those outlined in today’s training? | **⃝** | **⃝** | **⃝** | **⃝** | **⃝** |
| **12** | Manage a discussion with an OVER-CONTRIBUTING/VERBOSE LEARNER? | **⃝** | **⃝** | **⃝** | **⃝** | **⃝** |
| **13** | Manage a discussion with a QUIET/NON-ENGAGED LEARNER? | **⃝** | **⃝** | **⃝** | **⃝** | **⃝** |
| **14** | Manage a discussion with a HOSTILE/CONTRARY LEARNER? | **⃝** | **⃝** | **⃝** | **⃝** | **⃝** |

| After completing today’s training, how prepared do you feel to: | | | | | | |
| --- | --- | --- | --- | --- | --- | --- |
|  | | **Strongly Disagree** | **Disagree** | **Neutral** | **Agree** | **Strongly Agree** |
| **15** | The content of today’s training matched the learning objectives. | **⃝** | **⃝** | **⃝** | **⃝** | **⃝** |
| **16** | The group activities aided in my learning. | **⃝** | **⃝** | **⃝** | **⃝** | **⃝** |
| **17** | The facilitators were effective teachers. | **⃝** | **⃝** | **⃝** | **⃝** | **⃝** |
| **18** | Facilitate were responsive to our questions. | **⃝** | **⃝** | **⃝** | **⃝** | **⃝** |
| **19** | Our questions were answered clearly. | **⃝** | **⃝** | **⃝** | **⃝** | **⃝** |
| **20** | The length of today’s training was appropriate. | **⃝** | **⃝** | **⃝** | **⃝** | **⃝** |
| **21** | The organization of today’s training was effective. | **⃝** | **⃝** | **⃝** | **⃝** | **⃝** |

What is one lingering question you have after today's workshop? (We will collect these and send out the answers)

What was the most effective part of today's training that contributed to your learning?

In what ways should we consider changing this training to better prepare future learners?

What other comments or concerns do you have about your experience today?

**Part 2: Post Workshop Evaluation**

| PRIOR to today's workshop, how would you rate your confidence with your ability to: | | | | | | |
| --- | --- | --- | --- | --- | --- | --- |
|  | | Not Confident | Minimally Confident | Moderately Confident | Very Confident | Extremely Confident |
| **1.** | Recognize the various forms of patient-initiated identity-based harassment. | **⃝** | **⃝** | **⃝** | **⃝** | **⃝** |
| **2.** | Discuss the prevalence of patient-initiated identity-based harassment | **⃝** | **⃝** | **⃝** | **⃝** | **⃝** |
| **3.** | Discuss the impact of patient-initiated identity-based harassment. | **⃝** | **⃝** | **⃝** | **⃝** | **⃝** |
| **4.** | Identify barriers to responding to patient-initiated identity-based harassment. | **⃝** | **⃝** | **⃝** | **⃝** | **⃝** |
| **5.** | Describe the role of bystanders in addressing patient-initiated identity-based harassment. | **⃝** | **⃝** | **⃝** | **⃝** | **⃝** |
| **6.** | Describe three strategies for responding to identity-based harassment from patients. | **⃝** | **⃝** | **⃝** | **⃝** | **⃝** |

| AFTER today's workshop, how would you rate your confidence with your ability to: | | | | | | |
| --- | --- | --- | --- | --- | --- | --- |
|  | | Not Confident | Minimally Confident | Moderately Confident | Very Confident | Extremely Confident |
| **1.** | Recognize the various forms of patient-initiated identity-based harassment. | **⃝** | **⃝** | **⃝** | **⃝** | **⃝** |
| **2.** | Discuss the prevalence of patient-initiated identity-based harassment | **⃝** | **⃝** | **⃝** | **⃝** | **⃝** |
| **3.** | Discuss the impact of patient-initiated identity-based harassment. | **⃝** | **⃝** | **⃝** | **⃝** | **⃝** |
| **4.** | Identify barriers to responding to patient-initiated identity-based harassment. | **⃝** | **⃝** | **⃝** | **⃝** | **⃝** |
| **5.** | Describe the role of bystanders in addressing patient-initiated identity-based harassment. | **⃝** | **⃝** | **⃝** | **⃝** | **⃝** |
| **6.** | Describe three strategies for responding to identity-based harassment from patients. | **⃝** | **⃝** | **⃝** | **⃝** | **⃝** |

| How likely are you to use one or more of the strategies discussed today within the next two months? | | | | |
| --- | --- | --- | --- | --- |
| Very Unlikely | Somewhat Unlikely | Equally as Likely and Unlikely | Somewhat Likely | Very Likely |
| **⃝** | **⃝** | **⃝** | **⃝** | **⃝** |

What barriers or challenges do you anticipate may interfere with your ability to use those strategies?

|  | | | | | | |  |
| --- | --- | --- | --- | --- | --- | --- | --- |
| Please select the option that best matches your opinion with the following statements. | | | | | | | Pre select the option that best matches your opinion with the following statements. |
|  | | **Strongly Disagree** | **Somewhat Disagree** | **Neutral** | **Somewhat Agree** | **Strongly Agree** |  |
| **1.** | The content was relevant to my day-to-day work. | **⃝** | **⃝** | **⃝** | **⃝** | **⃝** |  |
| **2.** | The activities/examples were beneficial to my learning. | **⃝** | **⃝** | **⃝** | **⃝** | **⃝** |  |
| **3.** | The facilitators responded effectively to questions and concerns. | **⃝** | **⃝** | **⃝** | **⃝** | **⃝** |  |
| **4.** | The facilitators were effective teachers. | **⃝** | **⃝** | **⃝** | **⃝** | **⃝** |  |
| **5.** | I have learned new ways to think about issues addressed in this session. | **⃝** | **⃝** | **⃝** | **⃝** | **⃝** |  |
| **6.** | I have learned a new skill or information that I can apply in my work as a result of participating in this session. | **⃝** | **⃝** | **⃝** | **⃝** | **⃝** |  |
| **7.** | I have the confidence to apply this skill/information in my work. | **⃝** | **⃝** | **⃝** | **⃝** | **⃝** |  |
| **8.** | would recommend the workshop to my peers. | **⃝** | **⃝** | **⃝** | **⃝** | **⃝** |  |

What is your most important takeaway from today’s training? Why?

Please share 1-2 ways we could improve our training to better serve our learners.

**Part 3: 6-week Participant Follow-up Survey**

1. Since participating in the workshop...

|  | **⃝** | I have experienced or observed patient-initiated identity-based harassment |
| --- | --- | --- |
|  | **⃝** | I have not experienced or observed patient-initiated identity-based harassment |

1. Which of these describes your experience(s) (Select all that apply)?

| **⃝** | I have observed patient-initiated identity-based harassment |
| --- | --- |
| **⃝** | I have experienced patient-initiated identity-based harassment |

1. What type(s) of patient-initiated identity-based mistreatment did you experience (Select all that apply)?


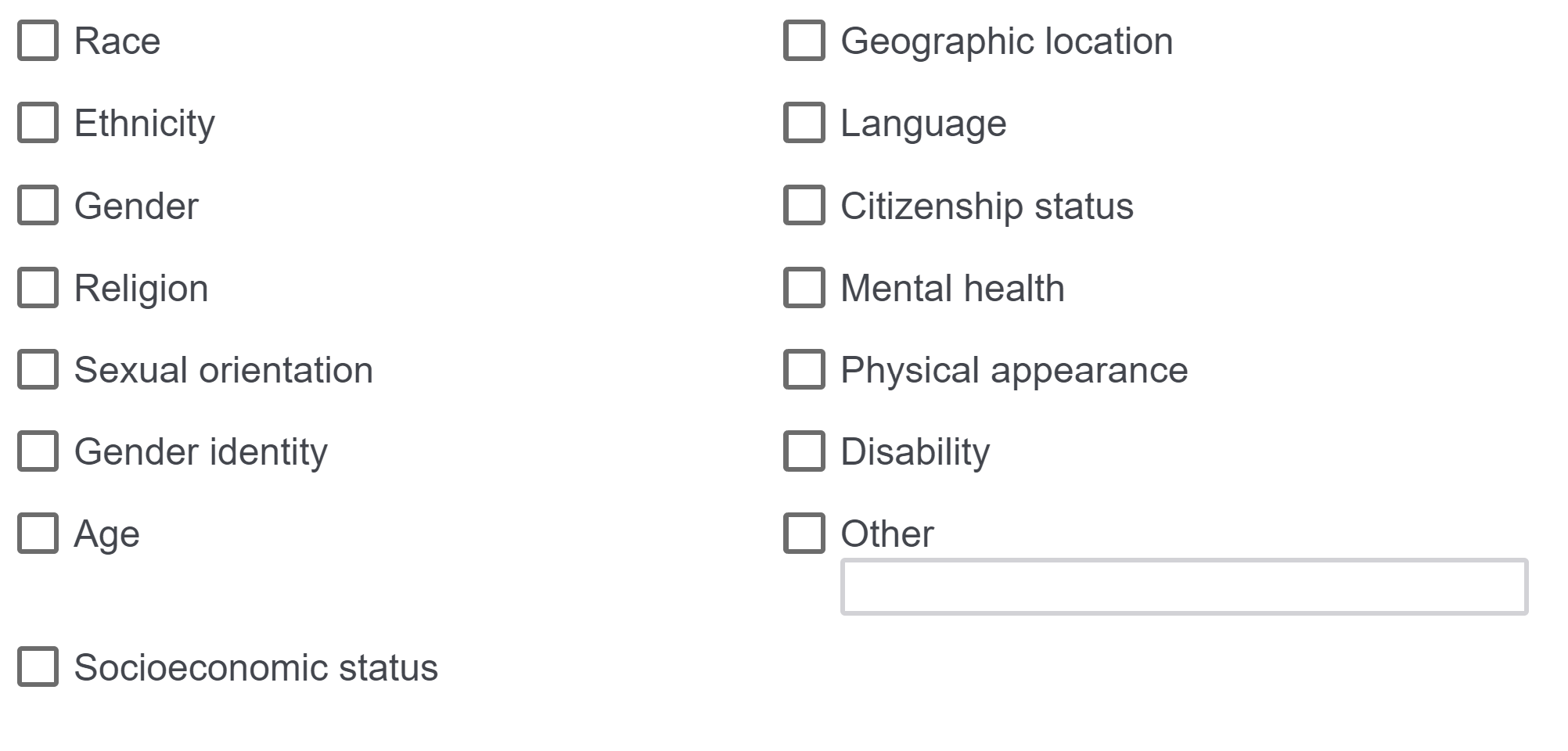


1. What type(s) of patient-initiated identity-based harassment did you observe (Select all that apply)?


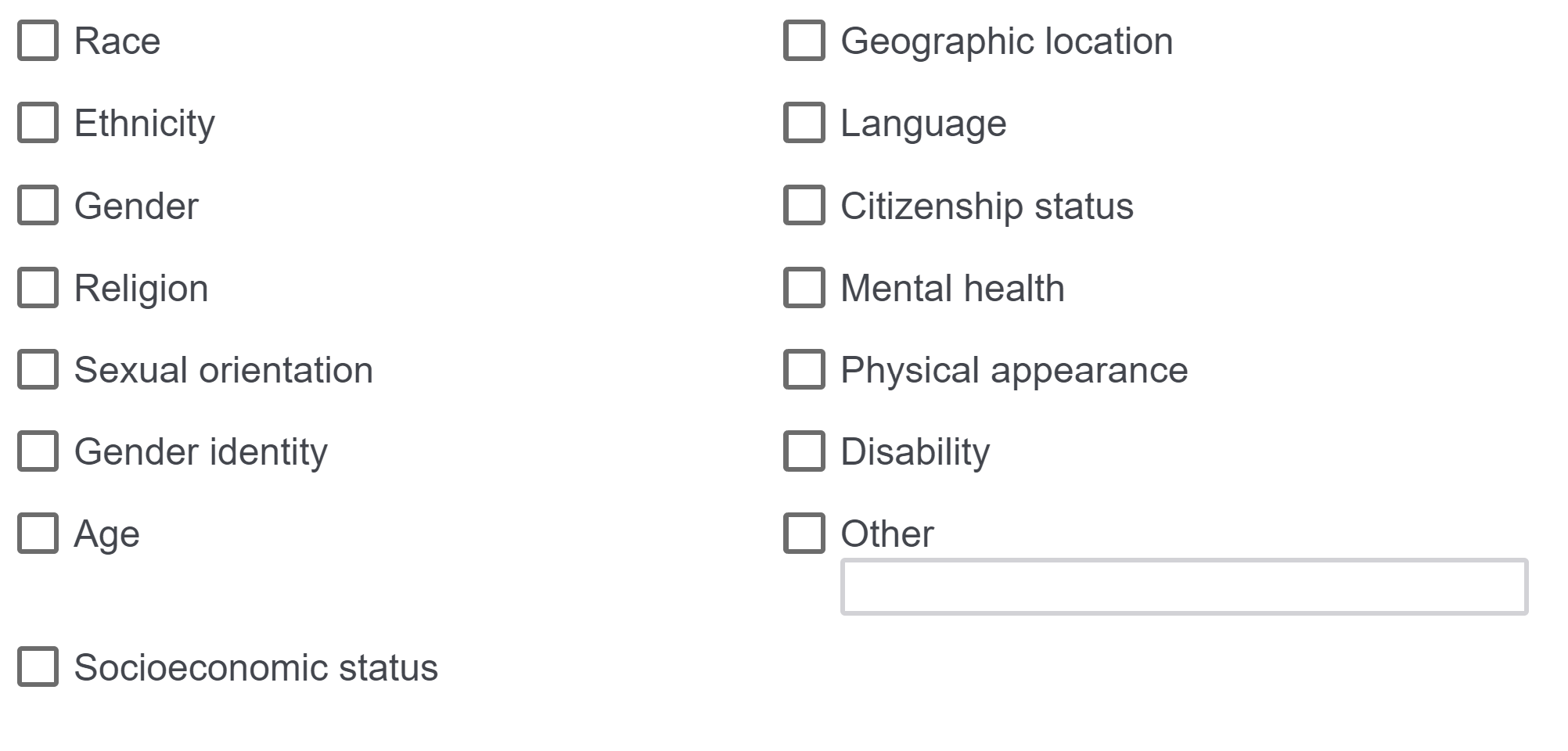


1. How often did these events occur

|  | | **Only Once** | **Monthly** | **Weekly** | **Daily or Almost** | **More than Once per Day** |
| --- | --- | --- | --- | --- | --- | --- |
|  | I have observed patient-initiated identity-based harassment | **⃝** | **⃝** | **⃝** | **⃝** | **⃝** |
|  | I have experienced patient-initiated identity-based harassment | **⃝** | **⃝** | **⃝** | **⃝** | **⃝** |

1. Did you ever directly address the harassment behavior(s) in the moment?

| **⃝** | Yes |
| --- | --- |
| **⃝** | No |
