# Supplementary material for: An Innovative Curriculum to Empower Trainees and Faculty to Address Patient-Initiated Identity-Based Misconduct in the Clinical Learning Environment
Source: MedEdPORTAL. 2026 Apr 9;22:11591. doi: 10.15766/mep_2374-8265.11591 (PMC13061878; doi:10.15766/mep_2374-8265.11591)
Supplement: Supplementary file 1 — I-RESPOND Toolkit.docxFacilitator Guide.docxEvaluations.docxPresentation.pptxScenario Scripts.docx [file mep_2374-8265.11591-s001.zip › E. Scenario Scripts.docx]

This appendix provides the scripted scenarios used for role-play and skills practice during the I-RESPOND workshop. The scenarios are intended to be read aloud or acted out and presented to the participants to practice applying the communication strategies from the I-RESPOND toolkit. Facilitators should select at least one or two scenarios to use during the workshop and may assign roles (e.g., resident, faculty, patient) to ensure participants experience different perspectives. Additional scenarios included here may be used for extended sessions or ongoing practice outside the initial workshop. Scenarios can also be adapted for departmental needs or specialty-specific contexts, while preserving the core focus on patient-initiated identity-based misconduct

**Scripts for responding to patient-initiated identity based harassment**

**Scenario 1**

Inappropriate / no response:

*An Asian-American nurse/medical student is trying to help a patient out of bed.*

**Patient yells:** Don't touch me! I only want American nurses who speak English to help me.

*The attending says nothing.*

Bystander correct response with debrief:

*An Asian-American nurse/medical student is trying to help a patient out of bed.*

**Patient yells:** Don't touch me! I only want American nurses who speak English to help me.

**Attending:** We ask that you treat all members of the team with respect. [Name] is an exceptional healthcare provider and we all work together as a team to provide you with the best care.

*Outside of the patient room or after the patient encounter*

**Attending:** I’m really sorry about how that patient behaved toward you. I want you to know that you have my support. How are you feeling?

**Student:** Thank you, that means a lot.

**Scenario 2**

Bystander responds out of turn (incorrect response):

*Student of color walks into room with white attending.*

*Patient stares at student.*

**Patient:** Where the hell are YOU from?

*Student looks like they are about to speak.*

**Attending** (interjects before student can speak): Don’t worry. He’s from here. He went to a good school, too.

Bystander allows learner to respond for self (correct response):

*Student of color walks into room with white attending.*

*Patient stares at student.*

**Patient:** Where the hell are YOU from?

**Student:** I’m sure you didn’t mean to be hurtful, but the way that question was phrased made me feel disrespected. Let’s focus on the topic at hand, which is your health/Let’s focus on your health and discuss what brings you in today.

**Scenario 3**

Person being harassed does not respond (incorrect):

*The female resident enters the room with the male attending.*

**Patient:** Oh, finally I get to see a doctor. This nurse over here keeps checking on me, and I’ve been asking when a real doctor will come. At least she’s pretty to look at.

*Attending and resident both say nothing.*

Person being harassed responds (correct):

*The female resident enters the room with the male attending.*

**Patient:** Oh, finally I get to see a doctor. This nurse over here keeps checking on me, and I’ve been asking when a real doctor will come. At least she’s pretty to look at.

**Female resident**: What I hear you say is that you think I am your nurse? Like I explained earlier, I am your doctor and would prefer for you to address me as Dr. Z.

**Scenario 4**

Inappropriate laughter:

*The patient is wheelchair/bed bound.*

*The medical student and the attending are in the room.*

*The attending instructs the medical student to auscultate the heart and lungs.*

**Attending:** Student doctor X, why don’t you go listen to the heart sounds and let me know what you hear?

**Student:** Mr. Brown, I’m going to listen to your heart now.

*Student leans over patient to accommodate wheelchair/bed.*

**Patient:** I’m going to pull you, so you fall into my lap.

*Attending laughs.*

Improved response:

*The patient is wheelchair/bed bound.*

*The medical student and the attending are in the room.*

*The attending instructs the medical student to auscultate the heart and lungs.*

**Attending:** Student doctor X, why don’t you go listen to the heart sounds and let me know what you hear?

**Student:** Mr. Brown, I’m going to listen to your heart now.

*Student leans over patient to accommodate wheelchair/bed.*

**Patient:** I’m going to pull you, so you fall into my lap.

**Attending:** Actually, student doctor X, why don’t you come stand over here while I listen? You can listen to the next patient.

*Attending directs student to stand on opposite side such that attending is between patient and student.*

**Attending:** Mr. Brown, that was not an appropriate way to talk to the student doctor and comments like that will not be tolerated. Let’s keep it professional and focus back on your health.
