# Supplementary material for: An Innovative Curriculum to Empower Trainees and Faculty to Address Patient-Initiated Identity-Based Misconduct in the Clinical Learning Environment
Source: MedEdPORTAL. 2026 Apr 9;22:11591. doi: 10.15766/mep_2374-8265.11591 (PMC13061878; doi:10.15766/mep_2374-8265.11591)
Supplement: Supplementary file 1 — I-RESPOND Toolkit.docxFacilitator Guide.docxEvaluations.docxPresentation.pptxScenario Scripts.docx [file mep_2374-8265.11591-s001.zip › A. I-RESPOND Toolkit.docx]

This handout is intended to be distributed directly to workshop participants during or immediately after the I-RESPOND curriculum session. It serves as a quick-reference guide for responding to patient-initiated identity-based misconduct in real time. The toolkit summarizes the key communication strategies introduced in the didactic and role-play exercises and is designed for practical use in the clinical setting (e.g., laminated badge card or pocket card). Facilitators should review the handout at the end of the workshop to reinforce learning and encourage participants to keep it accessible during clinical encounters.


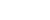

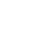

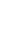


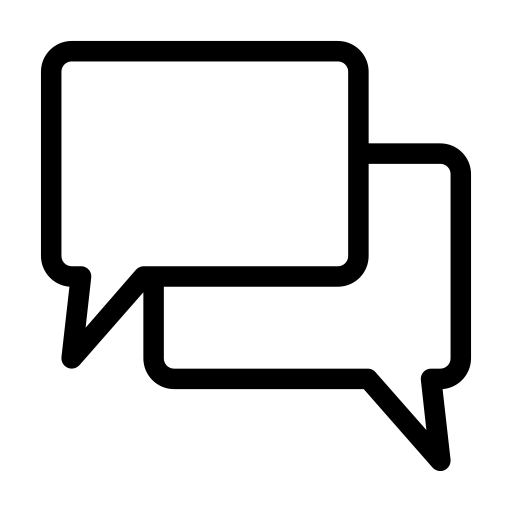


I-RESPOND TOOLKIT

Addressing patient-initiated

identity-based harassment

Resources

# GME Guidelines on Managing Patient Harassment

gme.medicine.uiowa.edu/diversity-related-initiatives-gme

# Office of Sexual Misconduct Response

osmrc.uiowa.edu/report-problem-0

# Office of Equal Opportunity and Diversity

diversity.uiowa.edu/office/equal-opportunity-and-diversity

IF **YOU ARE HARASSED** AND DECIDE TO RESPOND... IF **YOU OBSERVE HARASSMENT** OF A COLLEAGUE...

## Use “I” Statements

**Putting it all together**

**(example statement)**

“I’m sure you didn’t mean to be hurtful, but I feel uncomfortable when you comment

on my [appearance/ identity/background].

I want to give you the best care that I can so let’s keep our conversation professional.”


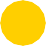


**I**

I feel uncomfortable when you comment on my [physical appearance/race/religion/age/etc.].”

## Repeat and Clarify Statement


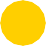


**R**

“Help me understand what you mean by that.” or “I heard you say . Will you clarify what you meant?”

## Emphasize Shared Goals


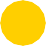


**E**

“I want to give you the best care that I can, but comments like that distract from my ability to focus on your care. Let’s keep our conversation professional.”

## Set Boundaries


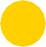


**S**

“Our hospital policy does not allow for discrimination on the base of [race/religion/gender/sexual orientation]. If you continue, I will have to leave the room.”

## Patient Actions Rather than Person


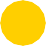


**P**

“I felt disrespected when you said that,” is less likely to make a harasser respond defensively than, “You are disrespectful.”

## Offer an Alternative


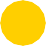


**O**

“I’d prefer if you call me ‘Doctor,’ rather than ‘baby’ or ‘honey’.”

## Separate INtent from Impact


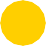


**N**

“I’m sure you didn’t mean to be hurtful when you said that, but it made me feel...”

## Don’t Use Humor


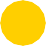


**D**

Use humor with caution as exaggeration or sarcasm may be misconstrued as reinforcement of prejudice.


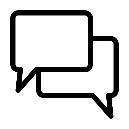


## Establish a culture of openness and respect

Expect that harassment will happen and recognize it when it occurs. “I wish that inappropriate comments and harassment by patients and visitors did not occur. But it does. I want to hear when things like this happen. It’s important that everyone feel safe and supported here.”


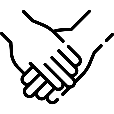


**Validate and offer support** “That was a difficult encounter. How are you doing?” “I want you to feel empowered to speak up

in a situation like that. You have my support.”


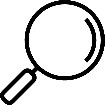


## Assess the situation

Does the person who was harassed appear uncomfortable or upset? Nonverbal cues should clue you in to whether the person desires help handling the situation.


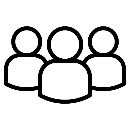


## Debrief with individual and team

“I would like to take some time to acknowledge and reflect

on how that encounter felt for

everyone.” “How do you think the encounter went? How can I/we address the situation differently next time to ensure a better outcome for everyone involved?


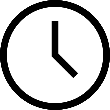


## Respond to the harassment in real time

“Mr. Z, we want to give you the best care and ask that you treat all of our team members with respect.” “We don’t tolerate that kind of language here, Let’s keep it professional.” Provide the harassed with an opportunity to leave the room.


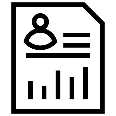


**Encourage reporting and documentation of patient harassment**

Create a written record of the incident.

- **I-RESPOND TOOLKIT Title icon:** Image made by inkubators from www.flaticon.com, retrieved from <https://www.flaticon.com/free-icon/conversation_5005217?term=speech+bubbles&page=1&position=17&origin=search&related_id=5005217> on August 9, 2023. Image is in public domain and free for personal and commercial purpose with attribution.
- **Establish a culture of openness and respect icon:** Image made by inkubators from www.flaticon.com, retrieved from <https://www.flaticon.com/free-icon/conversation_5005217?term=speech+bubbles&page=1&position=17&origin=search&related_id=5005217> on August 9, 2023. Image is in public domain and free for personal and commercial purpose with attribution.
- **Assess the situation icon:** Image made by phatplus from www.flaticon.com, retrieved from <https://www.flaticon.com/free-icon/zoom_900930?term=magnifying+glass&page=1&position=10&origin=search&related_id=900930> on August 9, 2023. Image is in public domain and free for personal and commercial purpose with attribution.
- **Respond to the harassment in real time icon:** Image made by Those Icons from www.flaticon.com, retrieved from <https://www.flaticon.com/free-icon/clock_2088617?term=clock&page=1&position=1&origin=search&related_id=2088617> on August 9, 2023. Image is in public domain and free for personal and commercial purpose with attribution.
- **Validate and offer support icon:** Image made by Freepik from www.flaticon.com, retrieved from <https://www.flaticon.com/free-icon/holding-hands_2059015?term=hand+holding&page=1&position=2&origin=search&related_id=2059015> on August 9, 2023. Image is in public domain and free for personal and commercial purpose with attribution.

## Debrief with individual and team icon: Image made by gungyoga04 from www.flaticon.com, retrieved from <https://www.flaticon.com/free-icon/team_11072062?term=group&page=1&position=7&origin=search&related_id=11072062> on August 9, 2023. Image is in public domain and free for personal and commercial purpose with attribution.

- **Encourage reporting and documentation of patient harassment icon:** Image made by Vectors Tank from www.flaticon.com, retrieved from [https://www.flaticon.com/free-icon/personnel-data_10191820?term=report+person&page= 1&position=50&origin=search&related_id=10191820](https://www.flaticon.com/free-icon/personnel-data_10191820?term=report+person&page=%20%201&position=50&origin=search&related_id=10191820) on August 9, 2023. Image is in public domain and free for personal and commercial purpose with attribution.
