# Supplementary material for: An Innovative Curriculum to Empower Trainees and Faculty to Address Patient-Initiated Identity-Based Misconduct in the Clinical Learning Environment
Source: MedEdPORTAL. 2026 Apr 9;22:11591. doi: 10.15766/mep_2374-8265.11591 (PMC13061878; doi:10.15766/mep_2374-8265.11591)
Supplement: Supplementary file 1 — I-RESPOND Toolkit.docxFacilitator Guide.docxEvaluations.docxPresentation.pptxScenario Scripts.docx [file mep_2374-8265.11591-s001.zip › _Educational Summary Report_11591.pdf]

MedEdPORTAL
THE AAMC JOURNAL OF TEACHING AND LEARNING RESOURCES

Original Publication Open Access

An Innovative Curriculum to Empower Trainees and Faculty to Address Patient-Initiated Identity-Based Misconduct in the Clinical Learning Environment

Nkanyezi Ferguson, MD, Lauren E. Hock, MD, Patrick Barlow, PhD, Aisha S. Jamison, MD*, Marcy E. Rosenbaum, PhD, Nicole Del Castillo, MD, MPH

*Corresponding author: ajamis@uw.edu

Abstract

Introduction: Training residents and attending physicians on effective communication strategies to manage biased patient and visitor comments is lacking. The I-RESPOND toolkit curriculum provides strategies for addressing identity-based misconduct in the clinical setting. **Methods:** Resident physicians and faculty in 12 departments at a single academic center participated in the workshop between June 2021 and February 2022. The workshop consisted of interactive didactics, an introduction to the I-RESPOND toolkit, and opportunities to practice communication strategies with formative feedback. Retrospective pre/postworkshop survey instruments and a follow-up survey were used to evaluate the workshop and subsequent experiences. **Results:** Sixty-six (32%) of 204 participants (including residents and attendings) completed the workshop evaluations, with 15 workshops facilitated. Both groups of participants were significantly more confident in their ability to respond to identity-based misconduct after participation. The retrospective pre/postworkshop analysis of their perceived change in confidence in addressing the workshop educational objectives showed a significant increase in median confidence score from pre- to postworkshop ($p < .001$). On the follow-up survey, participants' mean ± SD rating (disaggregated sample, 50 participants) for the likelihood of using at least one strategy in the next 2 months was 4.2 ± 1.01 (on a 5-point scale; 1 = *Very unlikely*, 5 = *Very likely*), with 9 (32%) of 28 participants indicating they had intervened in the moment to address the behavior. **Discussion:** This curriculum increased awareness of the impact of patient-initiated misconduct and helped inform institutional policies related to the management of disruptive discriminatory behavior from patients and visitors.

Keywords
Bias, Harassment, Case-Based Learning, Professionalism, Clinical Teaching/Bedside Teaching, Patient Initiated-Identity Based Harassment

Educational Objectives

By the end of this activity, learners will be able to:

1. Define and recognize the various forms of identity-based patient misconduct.
2. Discuss the prevalence and impact of patient misconduct on physicians.
3. Identify barriers to responding to incidents of misconduct.
4. Learn and apply at least 3 communication strategies for responding to patient misconduct.
5. Describe the role of bystanders in monitoring and responding to misconduct by patients.

Citation:
Ferguson N, Hock LE, Barlow P, Jamison AS, Rosenbaum ME, Del Castillo N. An innovative curriculum to empower trainees and faculty to address patient-initiated identity-based misconduct in the clinical learning environment. *MedEdPORTAL*. 2026;22:11591.
https://doi.org/10.15766/mep_2374-8265.11591

Introduction

Harassment, discrimination, and mistreatment based on a physician's identity group are prevalent, with patients and visitors being a common source. These incidents range from rude comments to explicit verbal and physical abuse. While both trainees and faculty broadly experience this mistreatment, residents are particularly at risk as they are often the first to interact with patients and have limited autonomy. As a result, harassment and mistreatment in the clinical learning environment negatively affect trainees' mental health, work performance, and ability to provide optimal patient care.[1-3] Attending physicians are meaningfully impacted as well. Gender-based harassment among attending physicians has been associated with poorer mental health, lower job satisfaction, decreased sense of

Copyright © 2026 Ferguson et al. This is an open-access publication distributed under the terms of the Creative Commons Attribution license.

safety at work, and increased turnover intentions.[4] Women physicians are disproportionately affected compared with men, as demonstrated in multiple studies conducted at large academic medical centers.[5] Ultimately, these encounters strain the patient–provider relationship and pose a threat to the well-being of both clinicians and patients.[4-6]

Identity characteristics that are commonly targeted include gender, race, ethnicity, sexual orientation, gender identity, and religion, with harassment based on gender being the most prevalent.[7,8] Patient and visitor misconduct ranges from inappropriate comments or innuendos to explicit verbal and physical abuse.[9] In a survey of 232 internal medicine residents, women, Black or Latinx, and Asian physicians more frequently reported biased behavior; 45% of Black or Latinx respondents experienced explicit epithets or rejection of care, and 87% of women reported sexual harassment.[10] Similarly, among more than 7,000 surgery residents, 32% reported experiencing gender identity discrimination, 30% reported experiencing verbal or physical abuse, 17% reported experiencing racial discrimination, and 10% reported experiencing sexual harassment.[7] These experiences extend beyond trainees, as surveys of resident and staff orthopedic surgeons indicate that patient-initiated discrimination and harassment are frequent and disproportionately affect female providers.[11]

Physicians who experience patient bias report significant emotional and professional consequences, including exhaustion, self-doubt, cynicism, and increased physician burnout.[9,12] Among both resident and staff orthopedic surgeons, patient-initiated discrimination and harassment have been shown to negatively affect the workplace environment and overall job satisfaction.[11] In addition to those directly targeted, nontargeted bystanders report moral distress and uncertainty about how to respond when witnessing these encounters.[12]

Despite these impacts, fewer than 5% of resident physicians formally report incidents of patient-initiated bias or harassment when they occur.[8,13,14] Furthermore, for resident physicians who wish to address patient or visitor misconduct, formal reporting mechanisms and educational frameworks for managing these difficult encounters are often lacking.[2,15,16] Additional barriers identified by both medical trainees and attending physicians include lack of response skills, insufficient support from senior colleagues and institutional leadership, and the perception that responding may have limited utility or carry professional risk.[9]

Educational interventions that prepare trainees and faculty to respond to all types of identity-based misconduct within the clinical learning environment are sorely needed. A few educational models have been described to help prepare resident physicians specifically to address these encounters. These varied approaches include constructive guidance, case-based scenarios, practical communication tools, and simulated patient training.[17-19] We propose an interactive training curriculum based on the I-RESPOND toolkit that provides opportunities for practice of communication skills and formative feedback. Additionally, the I-RESPOND toolkit offers a clear guide on what to say or do when encountering or witnessing misconduct. Among the published curricula,[17-19] most of the reported educational interventions have focused solely on residents or a single discipline.

Here, we provide a curriculum of communication strategies for responding to misconduct, grounded in established approaches.[20-24] The role-playing scenarios were used to help participants reinforce the skills presented at the workshop. We also emphasized the role of faculty and peer intervention in settings of harassment.[20] A practical communication skills I-RESPOND toolkit was introduced. The I-RESPOND toolkit and workshop includes several communication strategies, such as disclosing the recipient's personal feelings with "I" statements, addressing the behavior, clarifying the patient's comments, establishing a culture of respect while setting boundaries, and presenting an alternative perspective.[20,24] We implemented clinical video scenarios during the workshop to highlight concepts and skills introduced during the training to support individual needs for reinforcing concepts learned.

Through this curriculum, we intend to broaden our audience by reaching medical learners and faculty across institutional GME programs. The aim of this initiative is to increase trainee and faculty confidence and their ability to address patient and visitor harassment and misconduct as they occur. The GME leadership emphasized that the chosen time should reach the broadest possible audience of both trainees and faculty.

Methods

Curriculum Development

We collaborated with a multidisciplinary team of faculty and resident physicians from across GME at our institution to create the I-RESPOND toolkit and educational workshop. All team members had varying expertise in GME, diversity, equity, and inclusion management, curriculum development, and medical education research. We modeled our framework after the Six-Step Approach to Curriculum Development for Medical Education[25] and utilized an established logic model for effective program planning, collaboration, and monitoring (Figure).[26] The workshop and toolkit development followed 5 major stages:

Copyright © 2026 Ferguson et al. This is an open-access publication distributed under the terms of the Creative Commons Attribution license.

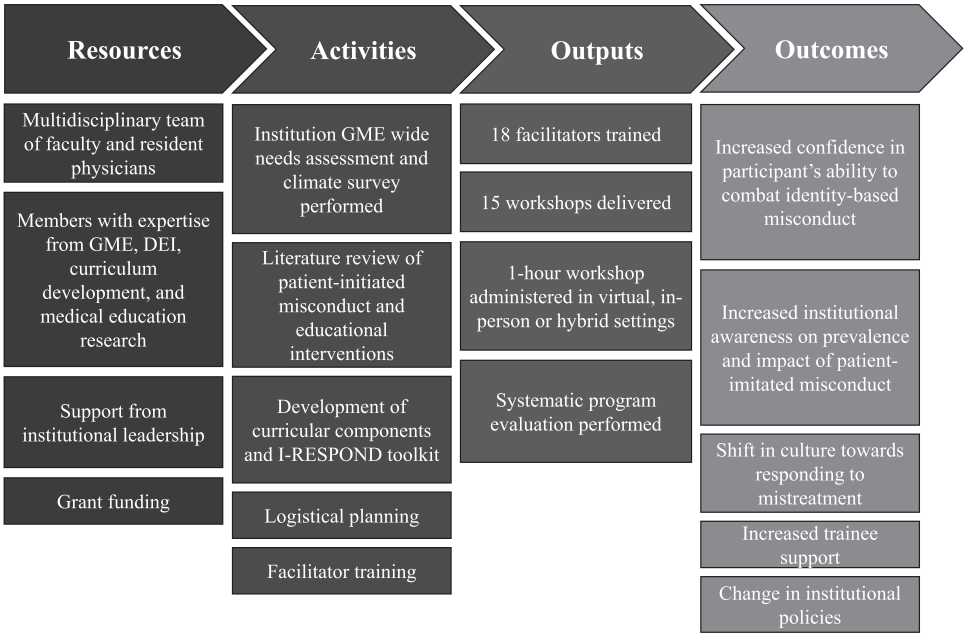

Figure. Logic model used to describe the I-RESPOND toolkit and patient-initiated harassment curriculum development process.

needs determination, curriculum development, facilitator training, implementation, and evaluation.

Prior to us developing the workshop, we conducted a climate survey and needs assessment of the GME trainees in 13 residency programs, using an online survey that had similar language and themes as those in a prior survey conducted by McKinley et al.[13] The climate survey respondents were primarily residents and fellows, whereas the workshop evaluation cohort included both residents and faculty across 12 departments. Our climate survey identified participants' existing knowledge of the prevalence, severity, sources, and impact of identity-based harassment during their clinical training. Additionally, our survey assessed trainees' confidence in responding to mistreatment from patients and visitors. From our results, qualitative and quantitative data were used to tailor the program's goals and objectives to the institution's needs.

During the curriculum development phase, we conducted an extensive literature review to inform curricular and toolkit development. We performed searches related to the incidence of patient-initiated identity-based harassment and misconduct toward physicians and educational programs, as well as approaches to managing this misconduct. Our content experts in communication then synthesized this information, integrated it into the workshop content, and provided a delivery approach. The toolkit of specific communication strategies for responding to identity-based misconduct was based on a framework of established approaches, tailored toward medical learners and faculty.[20-23,27] We established a timeline, format, target audience, implementation strategies, and evaluation approaches. The team also developed scripted video clinical scenarios and designed the I-RESPOND toolkit (Appendix A).

We then created training materials to ensure facilitators could effectively deliver the workshop curriculum (Appendix B). The facilitators, comprising GME faculty from various medical specialties, either applied or were nominated and selected through a competitive application process based on content expertise and facilitator experience. We administered a rigorous 4-hour training session, led by content experts using a train-the-trainer model, to a total of 18 faculty facilitators in efforts to ensure high quality, consistent, and reproducible delivery of the standardized curriculum material. The workshop facilitators received background literature and an introduction to the workshop materials. They were also trained in effective communication skills for delivering curricular content, providing formative feedback, and managing difficult conversations. Each facilitator's initial workshop was evaluated by an experienced facilitator, and concurrent feedback was provided (Appendix C). We provided ongoing facilitator development with a national communications expert, and we held debriefing sessions for facilitators to provide ongoing curricular feedback.

After curriculum development, we notified program directors during GME committee meetings and asked them to identify the

Copyright © 2026 Ferguson et al. This is an open-access publication distributed under the terms of the Creative Commons Attribution license.

optimal educational time within their departmental schedule (e.g., noon conference, morning conference, or grand rounds) that would maximize attendance.

Content Delivery

We began the workshop with an interactive didactic session that introduced definitions of identity groups, discrimination, harassment, and misconduct (Appendix D). We provided the resident and faculty physician participants with a review of the literature on patient-initiated misconduct and examples of de-identified harassing comments experienced by trainees (residents and fellows). This session also integrated polling questions not as part of the formal evaluation, but as an exercise designed to promote self-reflection and frame the workshop within participants' lived experiences. In the workshop, we emphasized the need for bystander faculty and peer support in situations of witnessed or reported patient misconduct.[20-23,27] We introduced the I-RESPOND toolkit (Appendix A) to provide a set of specific communication strategies for addressing identity-based misconduct in a nonjudgmental manner. Our presented strategies emphasized using "I" statements to express personal feelings[20] and depersonalizing patient's behaviors to minimize the act of making moral judgments.[20] Other methods included repeating and clarifying patient comments to facilitate further understanding and explanation of the patient's beliefs,[22,25] while setting boundaries to detail unacceptable behaviors.[20] Additional strategies include offering patients an alternative perspective or language,[28] emphasizing separating the intent of the mistreatment/misconduct from the impact,[28] and using humor with caution as it can sometimes reinforce prejudice.[24] Additionally, the toolkit offers stepwise guidance for bystanders who witness an individual experiencing patient misconduct. These strategies emphasize approaches for responding to harassment in real-time, assessing the situation,[24,28] establishing a culture of respect,[20,28] debriefing with the individual and team,[24] validating and offering support,[24] and encouraging formal documentation of the incident.[28]

We allowed the participants to apply these communication strategies to 2 interactive scripted video scenarios. We preformed and recorded the video scenarios using volunteer medical faculty and medical students following a curated script (Appendix E). We presented 2 scenarios (Scenarios 1 and 2) during the workshop, while additional scripts (Scenarios 3 and 4) were provided (Appendix E) for participants and facilitators to use as supplemental practice cases. We embedded these recordings into the presentation to illustrate key concepts (Appendix D, slide 29 and slide 37). In real time, the participants practiced using the I-RESPOND toolkit to address patient misconduct in clinical scenarios within both large-group and small-group settings, with formative feedback provided by the facilitators. We utilized the video scenarios to highlight concepts and introduce the use of skills during the training to support and reinforce concepts learned. Use of educational role-play has also been shown to foster empathy for the perspectives of the health care worker in each scenario.[29] In addition, we provided the participants with a pocket-sized card containing the I-RESPOND toolkit and badge cards to serve as a quick reference guide to be used during clinical encounters (Appendix A).

Evaluation Methods

We measured the workshop outcomes through program evaluation immediately following the workshop, utilizing retrospective pre/postworkshop survey instruments.[30] This single retrospective pre/postworkshop survey measured participants' preparedness to identify and respond to patient-initiated misconduct before versus after the workshop, with response ratings on a 5-point Likert scale (1 = *Not prepared*, 5 = *Extremely prepared*) (Appendix C). We later administered a follow-up survey to all participants 6–14 weeks after the workshops to assess their experiences with patient misconduct after the workshop and outcomes of these encounters. Our team analyzed the responses from pre- and postworkshop surveys separately by role, faculty, and resident physician.

The University of Iowa Institutional Review Board granted ethical approval for workshop implementation and evaluation and determined this project to be exempt from review.

Results

Fifteen workshops were delivered to resident physicians and faculty in 12 unique separate departments at a single academic center between June 2021 and February 2022. The workshops were delivered in virtual, in-person, or hybrid settings across GME programs at our institution.

Retrospective Pre/Postworkshop Survey Analysis

Sixty-six (32%) of 204 participants completed the postworkshop evaluation. Among these participants, 41 were residents and 21 faculty. The remaining 4 participants, representing nurse practitioners, physician assistants, and other department staff, were excluded from the final analysis. Both the residents and faculty reported a significant increase in confidence (rated on a 5-point Likert scale; 1 = *Not confident*, 5 = *Extremely confident*) in their ability to achieve all the workshop objectives (as detailed in the retrospective pre/postworkshop survey) after completing

Copyright © 2026 Ferguson et al. This is an open-access publication distributed under the terms of the Creative Commons Attribution license.

their training. Among residents, the proportion indicating *Extremely confident* and *Very confident* rose from a range of 22%–39% prior to the workshop to a range of 82%–90% after completion (Table 1). Among faculty, the proportion indicating *Extremely confident* and *Very confident* rose from a range of 5%–14% prior to the workshop to a range of 57%–86% after completion (Table 1).

Wilcoxon signed-rank tests were conducted to examine the statistical significance of these shifts in confidence levels within each role (Table 1). Among the residents who participated in the workshop, there was an increase of 1.0 point in the median total confidence score from preworkshop to postworkshop; all items showed statistically significant changes in median scores ($p < .001$). Among the attendings, there was an increase in median total confidence score from 1.0 point preworkshop to 2.0 points postworkshop; all items showed statistically significant changes in median scores ($p < .001$).

On the retrospective postworkshop survey, in rating their likelihood of using one or more of the strategies they learned in the workshop within the next 2 months, 45% of all participants (disaggregated sample) reported they were *Somewhat likely* and 41% reported they were *Very likely* to use what they learned in the workshop.

Follow-up Analysis

A subgroup analysis using the postworkshop survey and the follow-up survey administered 6–14 weeks after workshop attendance was conducted to assess the impact of our workshop on the participants' behaviors. Fifty participants (75%) who completed the postworkshop survey also completed the follow-up survey. This subgroup's mean ± SD rating for the likelihood they would use one or more strategies from the workshop in the next 2 months was 4.2 ± 1.01 (of 5 points). In the follow-up survey, 28 of the 50 matched participants had experienced or observed at least one instance of patient-initiated misconduct. Nineteen of those 28 reported observing patient-initiated misconduct, 20 reported experiencing patient-initiated misconduct, and 12 reported both observing and experiencing it. In the initial climate survey, 13% of respondents (34 of 262) reported personally experiencing harassment and took action in response. Additionally, 14% (38 of 262) said they witnessed harassment and intervened on someone else's behalf. In a follow-up survey conducted 6–14 weeks after the workshop, 32% of the 28 participants (9 individuals) reported having intervened in real time to address harassment at least once. Interestingly, these 9 respondents were equally likely to intervene whether they had personally experienced the harassment (7 people) or had only observed it happening to others (8 people). All 9 who intervened utilized at least one of the 8 strategies from the I-RESPOND toolkit, with the S – Set Boundaries strategy being the most frequently used.

Quality and Success Evaluation

In evaluating the quality and success of the workshop, our postworkshop survey showed that between 58% and 83% of resident respondents *Strongly agreed* with the 8 statements

Table 1. Participants' Perceived Confidence Prior to and After the Workshop on Addressing Identity-based Misconduct in the Clinical Setting ($N = 62$)

	Not/minimally confident n (%)		Moderately confident n (%)		Very/extremely confident n (%)		*Med* (IQR)[a]		
Item	Retro Pre	Post	Retro Pre	Post	Retro Pre	Post	Retro Pre	Post	*p*
Recognize the various forms of patient-initiated identity-based harassment.									
Trainee (*N* = 41)	6 (15)	1 (2)	19 (46)	5 (12)	16 (39)	35 (85)	3 (3, 4)	4 (4, 5)	<.001
Faculty (*N* = 21)	6 (29)	0 (0)	12 (57)	3 (14)	3 (14)	18 (86)	3 (2, 3)	4 (4, 4)	<.001
Discuss the prevalence of patient-initiated identity-based harassment.									
Trainee (*N* = 40)	14 (34)	2 (5)	17 (41)	5 (12)	10 (24)	33 (82)	3 (2, 3.5)	4 (4, 5)	<.001
Faculty (*N* = 21)	11(62)	0 (0)	7 (33)	9 (43)	1 (5)	12 (57)	2 (2, 3)	4 (3, 4)	<.001
Discuss the impact of patient-initiated identity-based harassment.									
Trainee (*N* = 41)	11 (27)	1 (2)	16 (39)	5 (12)	14 (34)	35 (85)	3 (2, 4)	4 (4, 5)	<.001
Faculty (*N* = 21)	11(52)	0 (0)	7 (33)	8 (38)	3 (14)	13 (62)	2 (2, 3)	4 (3, 4)	<.001
Identify barriers to responding to patient-initiated identity-based harassment.									
Trainee (*N* = 41)	10 (24)	1 (2)	19 (46)	3 (7)	12 (29)	37 (90)	3 (2.5, 4)	4 (4, 5)	<.001
Faculty (*N* = 21)	7 (33)	0 (0)	12 (57)	5 (24)	9 (9)	16 (76)	3 (2, 3)	4 (3.5, 4)	<.001
Describe the role of bystanders in addressing patient-initiated identity-based harassment.									
Trainee (*N* = 41)	18 (44)	1 (2)	11 (27)	5 (12)	12 (29)	35 (85)	3 (2, 4)	4 (4, 5)	<.001
Faculty (*N* = 21)	9 (43)	0 (0)	11 (52)	5 (24)	1 (5)	16 (76)	3 (2, 3)	4 (3.5, 4)	<.001
Describe three strategies for responding to identity-based harassment from patients.									
Trainee (*N* = 41)	20 (49)	1 (2)	12 (29)	4 (10)	9 (22)	36 (88)	3 (2, 3)	4 (4, 5)	<.001
Faculty (*N* = 21)	13 (62)	0 (0)	7 (33)	7 (33)	1 (5)	14 (67)	2 (2, 3)	4 (3, 4)	<.001

Abbreviations: IQR, interquartile range; Retro, retrospective.
[a]Rated on a 5-point scale (1 = *Not Confident,* 5 = *Extremely confident*).

Copyright © 2026 Ferguson et al. This is an open-access publication distributed under the terms of the Creative Commons Attribution license.

about the quality and content of the workshop (Table 2). Between 55% and 75% of the faculty *Strongly agreed* with the 8 statements about the quality and content of the workshop (Table 2). In addition, among all participants 57% *Strongly agreed* that they had the confidence to apply this information in their work (Table 2). Overall, in both roles, the median ratings for all 8 items were between 4 (*Agree*) and 5 (*Strongly agree*), with 4 being within the 25th percentile of responses and 5 being within the 75th percentile of responses.

Discussion

Literature has shown that for those who wish to address patient or visitor misconduct, reporting infrastructure and educational frameworks for managing these encounters are lacking.[2,15,16] Additional barriers reported by residents and faculty physicians include lack of response skills, insufficient support from senior colleagues and institutional leadership, and the perception that responding may have limited utility or carry professional risk.[9]

The I-RESPOND curriculum builds on prior educational approaches while also contributing to the literature on approaches to address harassment in GME. Unlike earlier training models that primarily focused on trainees, this program intentionally engaged both trainees and faculty across multiple specialties, recognizing faculty as essential responders and sources of support for resident physicians. By emphasizing faculty-supported bystander intervention through a train-the-trainer model and providing a concise, real-time clinical toolkit, I-RESPOND extends its impact beyond individual learners and promotes shared responsibility for addressing patient-initiated misconduct.

This curriculum highlighted and filled existing knowledge and skills gaps among faculty at this institution, underscoring the need for deliberate faculty development to ensure effective intervention and meaningful cultural change. Additionally, its integration into institutional policy reinforces its potential as a sustainable and scalable framework for addressing

Table 2. Participants' Responses on Overall Quality and Success of the Workshop ($N = 65$)

	n (%)[a]				
**Item**	**Strongly Disagree**	**Disagree**	**Neutral**	**Agree**	**Strongly Agree**
The content was relevant to my day-to-day work.					
Trainee (*N* = 40)	1 (2)	0 (0)	5 (12)	10 (25)	24 (60)
Faculty (*N* = 20)	0 (0)	0 (0)	0 (0)	5 (25)	15 (75)
All (*N* = 64)	1 (2)	0	5 (8)	19 (30)	39 (61)
The activities/examples were beneficial to my learning.					
Trainee (*N* = 41)	1 (2)	0 (0)	2 (5)	12 (29)	26 (63)
Faculty (*N* = 20)	0 (0)	0 (0)	0 (0)	5 (25)	15 (75)
All (*N* = 65)	1 (1)	0	2 (3)	19 (29)	43 (66)
The facilitators responded effectively to questions and concerns.					
Trainee (*N* = 41)	0 (0)	0 (0)	2 (5)	9 (22)	30 (73)
Faculty (*N* = 20)	0 (0)	0 (0)	1 (5)	4 (20)	15 (75)
All (*N* = 65)	0	0	3 (5)	14 (21)	48 (74)
The facilitators were effective teachers.					
Trainee (*N* = 41)	0 (0)	0 (0)	2 (5)	5 (12)	34 (83)
Faculty (*N* = 20)	0 (0)	0 (0)	1 (5)	6 (30)	13 (65)
All (*N* = 65)	0	0	3 (5)	14 (21)	48 (74)
I have learned new ways to think about issues addressed in this session.					
Trainee (*N* = 41)	1 (2)	0 (0)	2 (5)	11 (27)	27 (66)
Faculty (*N* = 20)	0 (0)	0 (0)	0 (0)	6 (30)	14 (70)
All (*N* = 65)	1 (1)	0	2 (3)	20 (31)	42 (65)
I have learned a new skill or information that I can apply in my work as a result of participating in this session.					
Trainee (*N* = 41)	1 (2)	0 (0)	1 (2)	11 (27)	28 (68)
Faculty (*N* = 20)	0 (0)	0 (0)	1 (5)	6 (30)	13 (65)
All (*N* = 65)	1 (1)	0	2 (3)	19 (29)	43 (66)
I have the confidence to apply this skill/information in my work.					
Trainee (*N* = 41)	0 (0)	0 (0)	3 (7)	14 (34)	24 (58)
Faculty (*N* = 20)	0 (0)	0 (0)	1 (5)	8 (40)	11 (55)
All (*N* = 65)	0	1 (1)	4 (6)	23 (35)	37 (57)
I would recommend the workshop to my peers.					
Trainee (*N* = 41)	1 (2)	0 (0)	5 (12)	9 (22)	26 (63)
Faculty (*N* = 20)	0 (0)	0 (0)	0 (0)	5 (25)	15 (75)
All (*N* = 65)	1 (1)	0	5 (8)	17 (26)	42 (65)

[a]Frequency of response based on the number of valid responses to each item.

Copyright © 2026 Ferguson et al. This is an open-access publication distributed under the terms of the Creative Commons Attribution license.

patient-initiated identity-based misconduct within the clinical learning environment.

After this workshop, resident physicians and faculty were significantly more confident in their ability to recognize, report, and address identity-based misconduct from patients. Most participants noted they were highly likely to use the strategies presented in the workshop, and on follow-up, most reported using at least one of the I-RESPOND strategies. When comparing the findings of the climate surveywith those of the follow-up survey, promising trends emerge; however, there remains room for enhancing participant's confidence in addressing these behaviors.

In developing and implementing the program, many challenges were encountered due to the evolving and dynamic situation during the COVID-19 pandemic. As a result, the workshops were delivered in varying formats, including in-person, virtual, and hybrid. The content delivery modality could impact participant engagement, attendance, and learning. The program evaluation did not separate outcomes by format, but future studies should explore this dimension more directly. Given the demanding nature of working in health care, many attendees faced time constraints due to patient care responsibilities. These varying formats of workshops, time constraints, and the demanding nature of health care can introduce confounding variables. To accommodate this, the workshops were limited to 1 hour and offered during protected didactic time. Since this program was initiated at a single academic institution, there is a potential for sampling and selection bias. The challenges faced in 1 institution may not apply to all residency programs at all institutions.

Additionally, the method of evaluation of this program relies on self-reported data from participants, introducing the potential for response bias. Participants may provide answers that they believe are socially desirable, which can affect the accuracy of the reported outcomes. The retrospective pre/postworkshop survey relies on participants' recall of their preparedness before the workshop, which can precipitate recall bias, impacting the accuracy of these retrospective assessments.

Though the 6–14 weeks follow-up survey provides some insight into posttraining experiences, participants with longer intervals may have had more opportunities to experience or observe misconduct. An extended follow-up could offer a more comprehensive understanding of the workshop's effectiveness over time. These biases may limit the validity and generalizability of our findings. During data extraction, free-text items were not included, and we were unable to explore why some participants reported being less likely to use I-RESPOND strategies.

The I-RESPOND curriculum provides a flexible toolkit that can be adapted to the realities of different clinical contexts and levels of learner agency. However, an additional consideration in this work is the power differential between residents and faculty in the clinical learning environment. While some scenarios presented to participants highlighted the supportive role of faculty bystanders, others emphasized residents' capacity to respond independently. By rotating through roles during the simulated practice, both groups could potentially appreciate the nuance of responding to misconduct at different levels of training. Future frameworks can be expanded to include more resident- and faculty-specific scenarios to address these different roles.

In the future, we aim to create online modules for ongoing education and organize in-person communication skills sessions with standardized patients. We also recognize that the length of the facilitator guide and the time commitment for facilitator training may limit adoption. Streamlined or modular versions of the training, including online formats, could enhance feasibility for broader dissemination. Finally, it is crucial to establish institutional policies, a formal reporting infrastructure, supportive environments, and mechanisms for addressing mistreatment barriers. This environment can mitigate burnout, boost self-confidence among medical trainees, and foster an inclusive clinical learning environment.

Implementing a more standardized approach to administering this workshop with an extended postworkshop evaluation period should be explored. Institutional policies, formal reporting infrastructure, supportive and inclusive environments, and removal of barriers to addressing mistreatment should be in place to optimize the effective management of these encounters. Fostering this environment can reduce the risk of burnout, encourage self-assurance among medical trainees, and support an inclusive clinical learning environment.

This novel curriculum helped to increase institutional awareness surrounding the prevalence and impact of patient-initiated misconduct. Although institutional awareness and culture were not directly measured in this study, the process of developing and implementing the curriculum through engaging GME leadership, training faculty facilitators, and involving multiple departments brought greater institutional attention to patient-initiated misconduct. This contributed to a shift in our institution's culture toward responding to patient misconduct in real time

Copyright © 2026 Ferguson et al. This is an open-access publication distributed under the terms of the Creative Commons Attribution license.

and providing trainee support. We highlight the successful development and implementation of an educational intervention curriculum that can serve as a model for other academic medical institutions.

Appendices

A. I-RESPOND Toolkit.docx

B. Facilitator Guide.docx

C. Evaluations.docx

D. Presentation.pptx

E. Scenario Scripts.docx

All appendices are peer reviewed as integral parts of the Original Publication.

Nkanyezi Ferguson, MD: Fellowship Director of Micrographic Dermatology Surgery and Dermatologic Oncology, Associate Professor of Clinical Dermatology, and Clinic Director of Micrographic Dermatology Surgery, Department of Dermatology, University of Missouri Health Care

Lauren E. Hock, MD: Assistant Professor of Ophthalmology, Glaucoma Service, Sidney Kimmel Medical College at Thomas Jefferson University

Patrick Barlow, PhD: Clinical Assistant Professor, Department of Internal Medicine, University of Iowa Hospital and Clinics, and Associate Director of Program Evaluation, Institute for Clinical & Translational Science, The University of Iowa

Aisha S. Jamison, MD: Third-Year Dermatology Resident Physician, Department of Dermatology, University of Washington Medicine; ORCID: https://orcid.org/0009-0004-6629-167X

Marcy E. Rosenbaum, PhD: Professor, Department of Family Medicine, University of Iowa Hospital and Clinics

Nicole Del Castillo, MD, MPH: Clinical Assistant Professor and Chief of Diversity, Equity, and Inclusion Officer, Carle Illinois College of Medicine

Acknowledgments

The authors wish to thank the University of Iowa Graduate Medical Education Office: Mark C. Wilson, MD, MPH, Hillary Chappo, MHA, and Megan Raitt; the Department of Medical Education at HCA Healthcare: Gerald Wickham, MA, EdD; the University of Iowa Department of Ophthalmology & Visual Sciences: Thomas A. Oetting, MD, Michael Abramoff, MD, PhD, Erin M. Shriver, MD, and Kelsey Hunold; the University of Iowa Hardin Library for the Health Sciences: Janna Lawrence, MLIS; the University of Iowa Carver College of Medicine: David Moser, PhD, Monique Galpin, and Destinee Soubannarath Gwee, MD; the University of California, Riverside School of Medicine: Denise Martinez, MD; and the University of Iowa Inclusive Education and Strategic Initiatives: Brianna Marcelo, MA.

Disclosures

None to report.

Funding/Support

This work is supported by the American Medical Association Accelerating Change in Medical Education Innovation Grant Program.

Prior Presentations

Ferguson N, Hock L. Responding to patient-initiated identity-based harassment: an innovative curriculum to empower residents and faculty to address harassment in clinical training. Presented at ChangeMedEd 2021; September 29-October 1, 2021; Virtual.

Jamison A, Ferguson N. The impact of a pilot workshop on addressing patient-initiated identity-based harassment and mistreatment in the clinical learning environment. Presented at: Association of Professors of Dermatology Annual Meeting the Dermatology Teachers Exchange Group; September 23, 2022; Chicago, IL.

Del Castillo N, Adibu F, Fernandez J. “What to say” responding to patient-initiated identity-based misconduct in the clinical learning environment. Presented at: AAMC GDI Professional Development Conference; March 2025; Columbus, OH.

Ethical Approval

The University of Iowa Institutional Review Board reviewed this project.

References

1. Fnais N, Soobiah C, Chen MH, et al. Misconduct and discrimination in medical training: a systematic review and meta-analysis. *Acad Med*. 2014;89(5):817-827. https://doi.org/10.1097/ACM.0000000000000200

2. Cabrera MT, Enyedi LB, Ding L, MacDonald SM. Sexual harassment in ophthalmology: a survey study. *Ophthalmology*. 2019;126(1):172-174. https://doi.org/10.1016/j.ophtha.2018.09.016

3. Willness CR, Steel P, Lee K. A meta-analysis of the antecedents and consequences of workplace sexual misconduct. *Personnel Psychol*. 2007;60(1):127-162. https://doi.org/10.1111/j.1744-6570.2007.00067.x

4. Vargas EA, Brassel ST, Cortina LM, Settles IH, Johnson TRB, Jagsi R. #MedToo: a large-scale examination of the incidence and impact of sexual harassment of physicians and other faculty at an academic medical center. *J Womens Health*. 2020;29(1):13-20. https://doi.org/10.1089/jwh.2019.7766

5. Scholcoff C, Farkas A, Machen JL, et al. Sexual harassment of female providers by patients: a qualitative study. *J Gen Intern Med*. 2020;35(10):2963-2968. https://doi.org/10.1007/s11606-020-06018-3

6. Jagsi R, Griffith KA, Jones R, Perumalswami CR, Ubel P, Stewart A. Sexual harassment and discrimination experiences of academic medical faculty. *JAMA*. 2016;315(19):2120-2121. https://doi.org/10.1001/jama.2016.2188

Copyright © 2026 Ferguson et al. This is an open-access publication distributed under the terms of the Creative Commons Attribution license.

7. Hu YY, Ellis RJ, Hewitt DB, et al. Discrimination, abuse, misconduct, and burnout in surgical residency training. *N Engl J Med*. 2019;381(18):1741-1752. https://doi.org/10.1056/NEJMsa1903759
8. Notaro E, Pascoe VL, Hippe DS, Lachance K, Shinohara MM, DeNiro KL. Prevalence of sexual misconduct and sexual assault from patient to provider among women in dermatology and across specialties. *Int J Womens Dermatol*. 2020;7(3):270-275. https://doi.org/10.1016/j.ijwd.2020.10.006
9. Wheeler M, de Bourmont S, Paul-Emile K, et al. Physician and trainee experiences with patient bias. *JAMA Intern Med*. 2019; 179(12):1678-1685. https://doi.org/10.1001/jamainternmed.2019.4122
10. de Bourmont SS, Burra A, Nouri SS, et al. Resident physician experiences with and responses to biased patients. *JAMA Netw Open*. 2020;3(11):e2021769. https://doi.org/10.1001/jamanetworkopen.2020.21769
11. Ryan S, Bozoghlian M, Lawler E, Patterson B. Patient initiated discrimination and harassment-a descriptive survey of experiences within a single academic department. *Iowa Orthop J*. 2023;43(1):1-6.
12. Dyrbye LN, West CP, Sinsky CA, et al. Physicians' experiences with mistreatment and discrimination by patients, families, and visitors and association with burnout. *JAMA Netw Open*. 2022; 5(5):e2213080. https://doi.org/10.1001/jamanetworkopen.2022.13080
13. McKinley SK, Wang LJ, Gartland RM, et al; Massachusetts General Hospital Gender Equity Task Force. "Yes, I'm the doctor": one department's approach to assessing and addressing gender-based discrimination in the modern medical training era. *Acad Med*. 2019;94(11):1691-1698. https://doi.org/10.1097/ACM.0000000000002845
14. Scruggs BA, Hock LE, Cabrera MT, et al. A U.S. survey of sexual harassment in ophthalmology training using a novel standardized scale. *J Acad Ophthalmol*. 2020;12(1):e27-e35. https://doi.org/10.1055/s-0040-1705092
15. Stevens LA. Responding to the difficult patient. *Bull Am Coll Surg*. 2010;95(5):12-15.
16. Cannarella Lorenzetti R, Jacques CHM, Donovan C, Cottrell S, Buck J. Managing difficult encounters: understanding physician, patient, and situational factors. *Am Fam Physician*. 2013;87(6):419-425.
17. Wilkins KM, Goldenberg MN, Cyrus KD. ERASE-ing patient mistreatment of trainees: faculty workshop. *MedEdPORTAL*. 2019;15:10865. https://doi.org/10.15766/mep_2374-8265.10865
18. Pang J, Navejar N, Sánchez JP. Mistreatment in residency: intervening with the REWIND communication tool. *MedEdPORTAL*. 2022;18:11245. https://doi.org/10.15766/mep_2374-8265.11245
19. Sandoval RS, Afolabi T, Said J, Dunleavy S, Chatterjee A, Ölveczky D. Building a tool kit for medical and dental students: addressing microaggressions and discrimination on the wards. *MedEdPORTAL*. 2020;16:10893. https://doi.org/10.15766/mep_2374-8265.10893
20. Wheeler DJ, Zapata J, Davis D, Chou C. Twelve tips for responding to microaggressions and overt discrimination: When the patient offends the learner. *Med Teach*. 2019;41(10): 1112-1117. https://doi.org/10.1080/0142159X.2018.1506097
21. Goldenberg MN, Cyrus KD, Wilkins KM. ERASE: a new framework for faculty to manage patient mistreatment of trainees. *Acad Psychiatry*. 2019;43(4):396-399. https://doi.org/10.1007/s40596-018-1011-6
22. Nadal KL. A guide to responding to microaggressions. *CUNY Forum*. 2014;2(1):71-76.
23. Alfandre D, Geppert C. Discriminatory and sexually inappropriate remarks from patients and their challenge to professionalism. *Am J Med*. 2019;132(11):1251-1253. https://doi.org/10.1016/j.amjmed.2019.03.034
24. Goodman DJ. *Promoting Diversity and Social Justice: Educating People from Privileged Groups*. Routledge; 2011.
25. Kern DE. Overview: a six-step approach to curriculum development. In: Thomas PA, Kern DE, Highes MT, Chen BY, eds. *Curriculum Development for Medical Education: A Six-Step Approach*. 3rd ed. Johns Hopkins University Press; 2015: chapter 1.
26. Kekahio W, Cicchinelli L, Lawton B, Brandon PR. *Logic Models: A Tool for Effective Program Planning, Collaboration, and Monitoring*. US Department of Education, Institute of Education Sciences, National Center for Education Evaluation and Regional Assistance, Regional Educational Laboratory Pacific; 2014. REL 2014–025. Accessed September 16, 2025. https://files.eric.ed.gov/fulltext/ED544779.pdf
27. Hock LE, Barlow PB, Scruggs BA, et al. Tools for responding to patient-initiated verbal sexual harassment: A workshop for trainees and faculty. *MedEdPORTAL*. 2021;17:11096. https://doi.org/10.15766/mep_2374-8265.11096
28. Shankar M, Albert T, Yee N, Overland M. Approaches for residents to address problematic patient behavior: before, during, and after the clinical encounter. *J Grad Med Educ*. 2019;11(4): 371-374. https://doi.org/10.4300/JGME-D-19-00075.1
29. Bosse HM, Schultz JH, Nickel M, et al. The effect of using standardized patients or peer role play on ratings of undergraduate communication training: a randomized controlled trial. *Patient Educ Couns*. 2012;87(3):300-306. https://doi.org/10.1016/j.pec.2011.10.007
30. Pratt CC, McGuigan WM, Katzev AR. Measuring program outcomes: using retrospective pretest methodology. *Am J Eval*. 2000;21(3):341-349. https://doi.org/10.1177/109821400002100305

Received: March 14, 2025
Accepted: February 6, 2026
Published: April 9, 2026

Copyright © 2026 Ferguson et al. This is an open-access publication distributed under the terms of the Creative Commons Attribution license.
